# In silico exploration of natural xanthone derivatives as potential inhibitors of severe acute respiratory syndrome coronavirus 2 (SARS-CoV-2) replication and cellular entry

**DOI:** 10.1007/s10822-025-00585-5

**Published:** 2025-02-17

**Authors:** Vincent A. Obakachi, Vaderament-A. Nchiozem-Ngnitedem, Krishna K. Govender, Penny P. Govender

**Affiliations:** 1 Department of Chemical Sciences, University of Johannesburg, Doornfontein Campus, P. O. Box 17011, Johannesburg, 2028 South Africa; 2 Institut für Chemie, Universität Potsdam, Karl-Liebknecht-Str. 24-25, D-14476 Potsdam-Golm, Germany

**Keywords:** ACE2 inhibition, SARS-CoV-2 anti-viral therapy, Xanthone derivatives, Molecular docking and dynamics, MM/GBSA binding energy, Electrostatic potential (MEP) analysis

## Abstract

The COVID-19 pandemic, caused by SARS-CoV-2, has underscored the urgent need for effective antiviral therapies, particularly against vaccine-resistant variants. This study investigates natural xanthone derivatives as potential inhibitors of the ACE2 receptor, a critical entry point for the virus. We computationally evaluated 91 xanthone compounds derived from *Swertia chirayita*, identifying two promising candidates: 8-O-[β-D-Xylopyranosyl-(1→6)-β-D-glucopyranosyl]-1,7-dihydroxy-3-methoxy xanthone (XAN71) and 8-O-[β-D-Xylopyranosyl-(1→6)-β-D-glucopyranosyl]-1-hydroxy-3,7-dimethoxy-xanthone (XAN72). Molecular docking and dynamics simulations (MDDS) were performed to assess their binding energy and stability within the ACE2 active site, comparing them to the reference inhibitor MLN-4067. The top six compounds were selected based on their docking performance, followed by Molecular Mechanics/Poisson-Boltzmann Surface Area (MM/PBSA) calculations to quantify binding affinities. Additionally, molecular electrostatic potential (MEP) analysis was conducted to visualize electron density regions relevant to binding interactions. Our results demonstrate that XAN71 and XAN72 exhibit superior binding affinities of -70.97 and − 69.85 kcal/mol, respectively, outperforming MLN-4067 (-61.33 kcal/mol). MD simulations revealed stable interactions with key ACE2 residues, primarily through hydrogen bonds and hydrophobic contacts. The Molecular Electrostatic Potential(MEP) analysis further elucidated critical electron density regions that enhance binding stability. This study establishes XAN71 and XAN72 as viable candidates for ACE2 inhibition, providing a structural basis for their development as natural xanthone-based therapeutics against SARS-CoV-2. These findings highlight the potential of targeting ACE2 with natural compounds to combat COVID-19, particularly in light of emerging viral variants.

**Supplementary Information:**

The online version contains supplementary material available at 10.1007/s10822-025-00585-5.

## Highlights

- XAN71 and XAN72 exhibit stronger ACE2 binding than the reference inhibitor MLN-4067.
- XAN71 and XAN72 form unique and stable hydrogen bonds with ACE2 active site residues.
- Both ligands display stable RMSD values throughout 200 ns molecular dynamics simulations.
- Electrostatics and van der Waals interactions significantly enhance XAN71 and XAN72 binding.
- XAN72’s smaller HOMO-LUMO gap suggests higher reactivity compared to other compounds.

**Graphical abstract:**

**Supplementary Information:**

The online version contains supplementary material available at 10.1007/s10822-025-00585-5.

## Graphical abstract

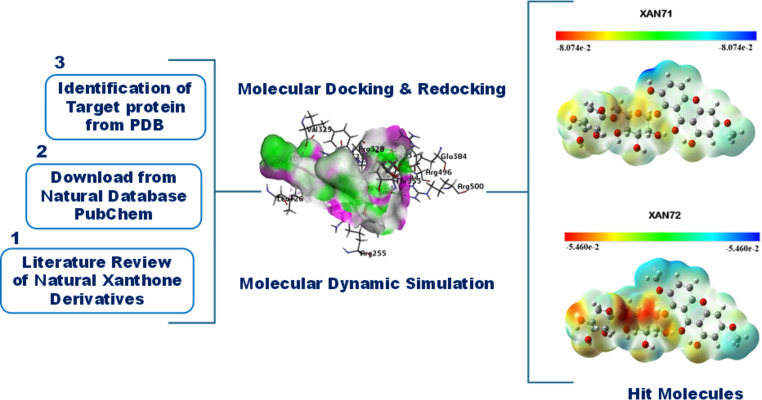

## Introduction

The global Coronavirus disease 2019 (COVID-19) pandemic, caused by the severe acute respiratory syndrome coronavirus 2 (SARS-CoV-2) virus, has had far-reaching implications on public health, economic stability, and societal structure [1]. As of September 8, 2024, the cumulative number of confirmed COVID-19 issues globally had reached a staggering 776 million, with over 103 million cases reported in the United States and 99.4 million in China alone [2]. Despite aggressive efforts in vaccination campaigns, the virus continues to evolve, leading to an increase in breakthrough infections and the development of vaccine-resistant variants. This persistence highlights the need for novel therapeutic strategies, particularly targeting critical viral entry points such as ACE2, a primary receptor for SARS-CoV-2 [3]. Recent studies have explored the potential of natural compounds, particularly xanthone derivatives, as inhibitors of ACE2, offering a promising avenue for anti-viral drug development against COVID-19. Xanthones, naturally occurring compounds in various plant sources, have demonstrated potent anti-viral activity against multiple pathogens, including coronaviruses. Their anti-viral properties are primarily attributed to their ability to disrupt viral replication and cellular entry processes [4, 5]. Considering SARS-CoV-2, xanthones are believed to block the interaction between the ACE2 receptor and the viral spike protein, which is essential for the infection of human cells by the virus [5, 6]. This inhibition can significantly reduce viral entry, thus mitigating the severity of COVID-19. Therefore, exploring xanthones as ACE2 inhibitors provides a new dimension to anti-viral therapy and opens the potential for plant-derived compounds in addressing pandemics.

The role of ACE2 as a therapeutic target has gained significant attention, especially considering the virus’s ability to exploit this receptor to gain entry into human cells. The ACE2 is a crucial regulator in the renin-angiotensin system (RAS) and a critical facilitator of viral attachment and infection [7]. Though adequate to an extent, current anti-viral therapies and vaccines face challenges due to viral mutations that reduce their efficacy. For instance, the waning protection of first-generation vaccines and the emergence of monoclonal antibody-resistant strains have prompted researchers to explore ACE2 inhibitors as a novel intervention [3].

Given the urgency to develop novel therapeutic approaches, natural inhibitors of ACE2, particularly xanthone derivatives, present a sustainable and accessible alternative to synthetic drugs. Xanthones are prevalent in medicinal plants from families such as Polygalaceae, Moraceae, Gentianaceae, and Guttiferae, which have been utilized in traditional medicine for centuries [8–10]. Their broad-spectrum anti-viral activity and relatively low toxicity make them compelling candidates for advancing COVID-19 management [4, 11]. Studies indicate that these compounds inhibit viral entry and modulate the immune response, crucial for controlling inflammation and preventing severe disease [12]. Previous studies have identified several xanthone derivatives that show promise as inhibitors of ACE2, making them potential candidates for the treatment of COVID-19 [8–11, 13–21]. As the pandemic continues to pose significant challenges to global health systems, investigating natural compounds with anti-viral properties can complement existing therapeutic strategies and provide new hope for managing emerging SARS-CoV-2 variants. Continued research into the molecular mechanisms fundamental to the anti-viral effects of these xanthones will be crucial for developing effective and scalable treatments for COVID-19. Given these findings, natural xanthone derivatives like XAN71 and XAN72 have emerged as promising candidates in this study. Both compounds demonstrated superior docking scores and binding affinities, outperforming other derivatives and the reference inhibitor MLN-4760. Their stable interactions with key ACE2 residues, supported by MD simulations, highlighted their structural compatibility and inhibitory potential. These attributes justify their prioritization for further investigation as potential antiviral agents targeting SARS-CoV-2. By leveraging plant-derived compounds with antiviral properties and the power of computational methodologies [22–24], this research offers a sustainable and accessible avenue for advancing COVID-19 management amidst evolving challenges.

## Materials and methods

### Preparation of the target protein, ACE2

The extra-cellular domain (ECD) of the ACE2 receptor, comprising 615 amino acid residues, is responsible for its catalytic activity. This domain includes the peptidase (PD), essential for interacting with the SARS-CoV-2 spike protein. It houses the active site where enzymatic processing occurs and serves as the critical region for direct interaction with the viral spike protein, facilitating the virus’s entry into host cells [25, 26].

**Fig. 1:**
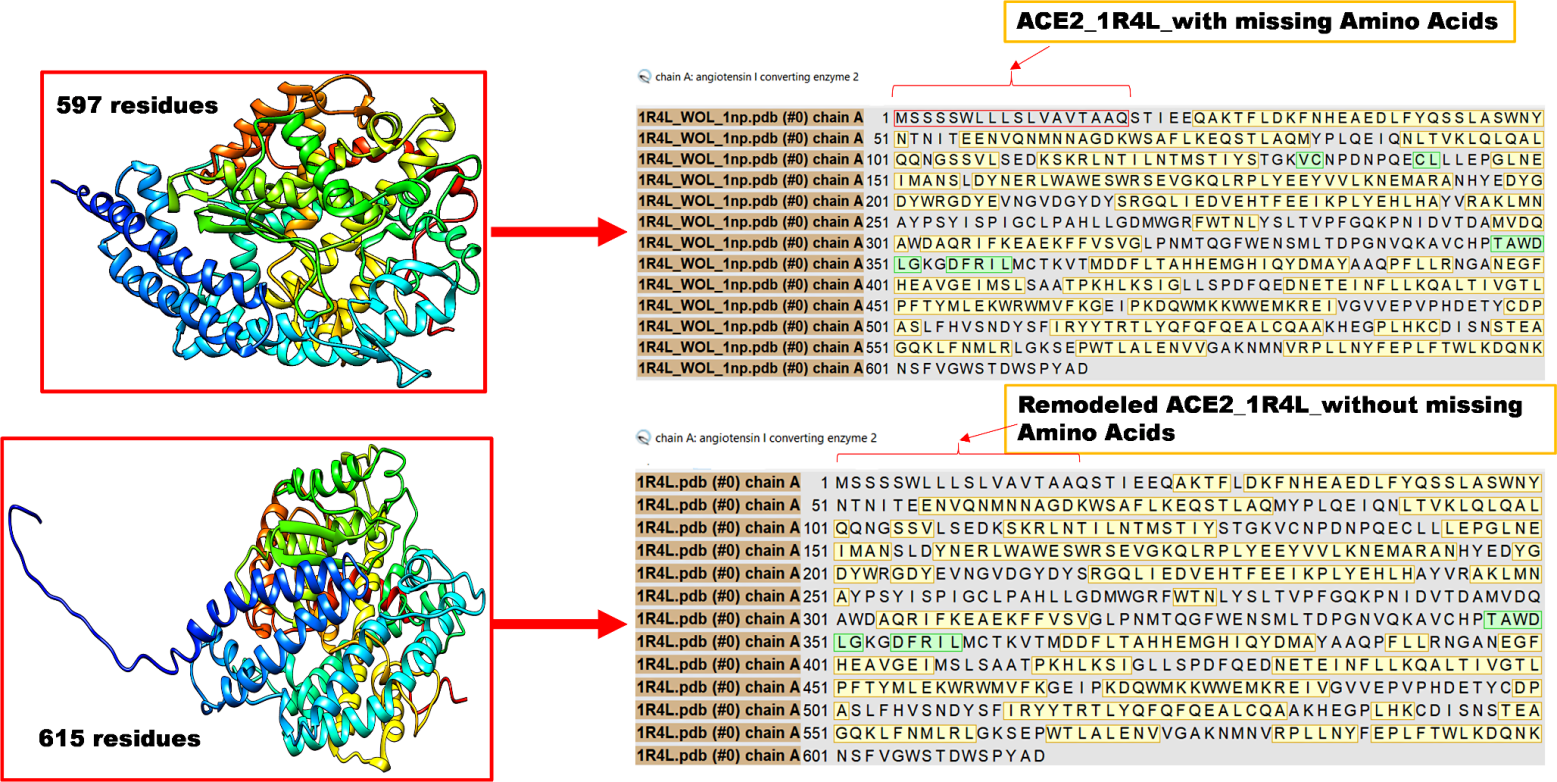
Remodeled ACE2 protein structure

To conduct accurate docking studies, preparing the target protein, ACE2, was essential to ensure it was optimal for ligand binding. Protein Acquisition was carried out by obtaining the crystal structure of human ACE2 from the Protein Data Bank (PDB) (https://www.rscb.org) [27]. The selected PDB entry has PDB ID: 1R4L, which represents the ACE2 protein in complex with the potent blocker MLN-4760 ((S, S)-2-[1-carboxy-2-[3-(3,5-dichlorobenzene)-3 H-imidazol4-yl]-ethylamino]-4-methyl pentanoic acid) [27]. In the Protein Preparation Wizard of Glide (Schrödinger Suite 2023-2) [28], bond orders were specified, hydrogens were added, disulfide bonds were formed, and water molecules were eliminated. Additionally, Prime was used to complete 18 missing amino acids and absent side chains and loops [28]. This resulted in unfolding at the tail end of the loop of the ACE2 protein, as shown in Fig. 1. However, this process is part of the conformational changes that ACE2 undergoes upon binding to the virus, which may involve proteolytic cleavage and subsequent structural alterations. Such unfolding is not only expected but is also crucial for facilitating viral entry into host cells [29]. The structure was then reduced using OPLS4 [28] and optimization of the hydrogen network to ensure the accurate orientation of side chains and protonation states. PROPKA [28] was used to predict the protonation states of titratable residues at a physiological pH of 7.4. A receptor grid file was generated to define the ligand-binding site, and the protein structure underwent restrained energy minimization using the OPLS4 force field. This process reduced steric clashes and optimized the protein conformation for stability.

### Selection of active site and grid generation

The binding pocket site of ACE2 was defined based on the binding location of the prototype inhibitors (MLN-4067). A grid was then generated around the binding pocket (Grid center: 40.61, 5.82, and 27.85. Innerbox:10, 10, and 10. Outbox: 25, 25, and 25) using the Receptor Grid Generation tool within Schrödinger Suite 2023-2, which provided a specific area for ligand docking, thus improving computational efficiency and accuracy. The van der Waals (vdW) radius scaling factor for non-polar atoms in the receptor was set to 1.0, with a partial charge threshold applied at 0.25. The docked ligand was confined to the enclosed box centroid of the Workspace ligand selected in the Receptor tab. At the same time, the size of the docked ligands was adjusted to 35 Å due to the length of our natural product ligands [28].

### Validation of prepared protein

**Fig. 2:**
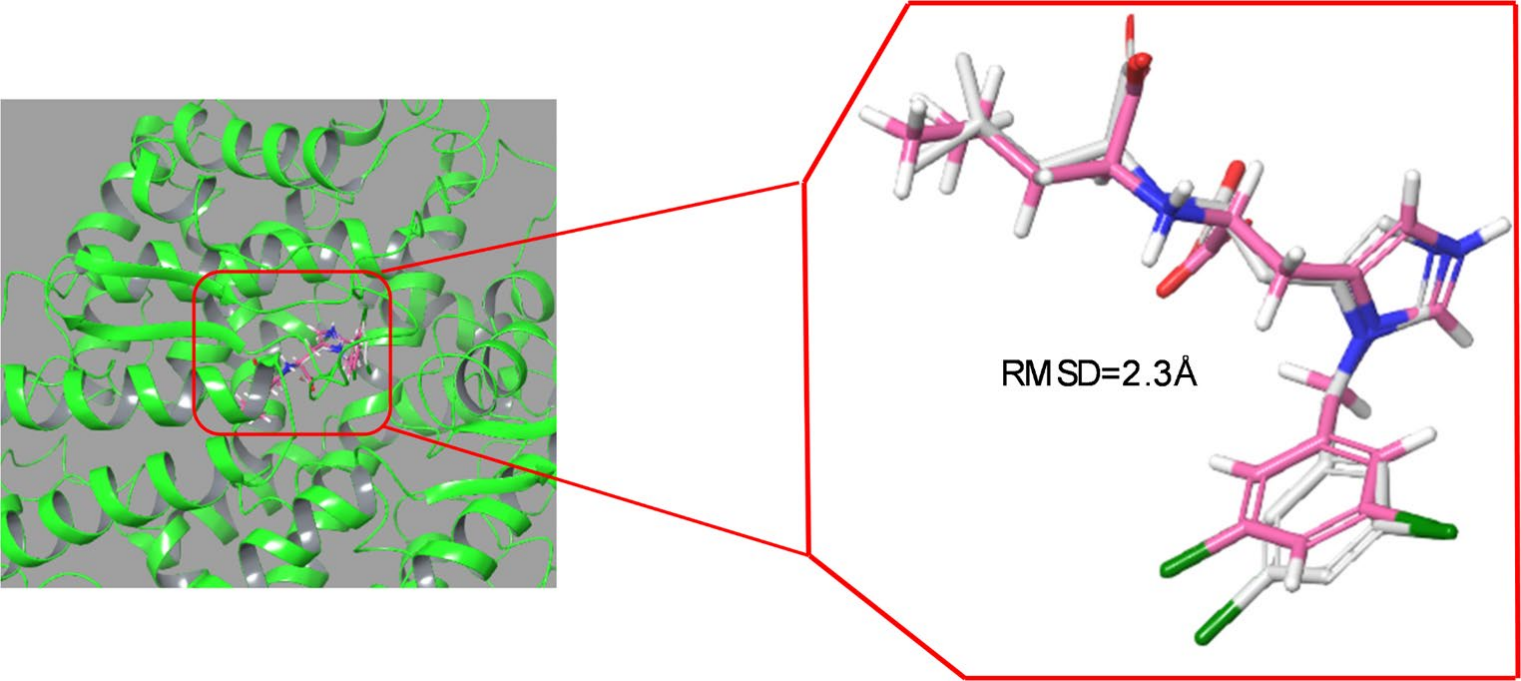
The re-docked pose of MLN-4067 (pink-carbons in thick tube model) with the original pose of original prototype ligand MLN-4067 extracted from the PDB (white-carbons in thick tube model) in the protein binding site. Amino acid residues and non-polar hydrogen atoms were hidden for clarity

The prepared protein structure was visually inspected to ensure no unresolved steric clashes or abnormal configurations. The protein’s binding site was validated by re-docking prototype ligands MLN-4067 to confirm the accuracy of the preparation. The docked pose of MLN-4067 (depicted with pink carbons in a thick tube model) was superimposed with the original prototype pose of MLN-4067 from the PDB (white carbons in a thick tube model) within the protein’s binding site. Non-polar hydrogen atoms and amino acid interactions were hidden for clarity in Fig. 2.

### Ligand preparation

The preparation of 91 xanthone derivatives (Table S1 in the supplementary material contains the compound names, sources, activities, and references) was carried out to ensure they were optimal for molecular docking studies. The chemical structures of the xanthone derivatives were downloaded in structure-data file (SDF) format from the NCBI PubChem database (https://pubchem.ncbi.nlm.nih.gov/). Ligand preparation was carried out to assign accurate bond orders and generate three-dimensional geometries. This was done in Maestro, Schrödinger Suite 2023-2, using LigPrep with the OPLS4 force field. The ionization states were also adjusted to pH 7.0 ± 2.0 using Epik 2. At most, 2–5 tautomers were generated for each ligand, and the compounds eventually amounted to 237.

### Molecular docking

Molecular docking was performed using the previously prepared receptor grid file in Schrödinger Maestro 2023-2 with the Glide tool. The Standard Precision (SP) setting was utilized for docking, with ligand sampling configured to be “flexible” to allow conformational changes. A partial charge cut-off of 0.15 was applied to ligand atoms, and the van der Waals radius scaling factor was adjusted to 0.80. For refinement, Gaussian 16 [30] was used to optimize the equilibrium geometries of the top-binding candidates. Optimization and frequency calculations were conducted using the B3LYP/6–31 + + G(d, p) level of theory and basis set, recognized for its accuracy in comparable studies [31]. Following this, the top docked ligands were re-docked to validate further and fine-tune their binding poses.

### Molecular dynamics (MD) simulation procedure

The ACE2-xanthone derivative complexes with the highest binding scores and the prototype ligand complex and unbound/free protein (Apo) were prepared for molecular dynamics (MD) simulations using Amber20 [32] with GPU acceleration. The FF14SB force field [33] was applied to ensure accurate modeling of the systems [34]. Ligand atomic partial charges were assigned in ANTECHAMBER using the General Amber Force Field (GAFF) combined with Restrained Electrostatic Potential (RESP) algorithms [35]. Using tLeaP, hydrogen atoms were added to the protein residues, and counter ions (Cl⁻ and Na⁺) were introduced to neutralize the system. The complexes were then solvated with TIP3P water in a cubic box, extending 10 Å beyond the protein atoms. The ligand-bound and unbound/free protein systems were initially minimized by performing 1000 steps of steepest descent, followed by 1000 steps of conjugate gradient minimization, with a 500 kcal mol⁻¹ Å⁻² force constant applied to restrain protein atoms. This initial minimization phase was tailed by an additional 1000 steps of each minimization method to refine the systems further, reducing potential energy and optimizing structural stability before (MD) simulation. The systems were gradually heated to 300 K over 50 ps under constant volume (NVT) conditions, applying weak positional restraints of 10 kcal mol⁻¹ Å⁻² to the protein atoms. Next, equilibration was performed at 300 K for 500 ps under constant pressure (NPT) conditions, maintaining a pressure of 1 atm via isotropic scaling. Temperature control was achieved using Langevin Dynamics [36] with a collision frequency of 1 ps⁻¹ to ensure stability throughout the simulation. The particle-mesh Ewald (PME) method was used to handle long-range electrostatic interactions, ensuring accurate computation of forces across periodic boundaries. A 10 Å cut-off was applied to limit calculations within a specified distance for short-range non-bonded interactions. The SHAKE algorithm [37] was used to constrain all hydrogen-involved bond lengths, enabling a larger integration time step for enhanced simulation efficiency without sacrificing accuracy. The MD simulation was conducted over 200 ns with a 2 fs time step, and simulation results were analyzed using the cpptraj program in the Amber 20 package.

### Post-MD simulation

After the (MD) simulation, the stability and structural dynamics of the protein-ligand complexes were analyzed using the AMBER 20 software package. The CPPTRAJ module computed essential structural parameters, including root mean square deviation (RMSD), radius of gyration (RoG), root mean square fluctuation (RMSF), and per residue energy decomposition (PRED), offering insights into the conformational stability throughout the simulation. The raw data were processed and visualized in OriginPro [38] to generate plots to interpret structural stability and flexibility trends.

### Binding energy calculation using MM/PBSA

Binding energy assessments were conducted using the Molecular Mechanics/Poisson–Boltzmann Surface Area (MM/PBSA) [39] approach to evaluate and compare the binding affinities of the protein-ligand complexes. This approach allows for the decomposition of free energy contributions from molecular interactions and solvation effects. The binding energy was computed by averaging over start frame 1 to end frame 20,000 at interval = 100 extracted from the 200 ns (MD) simulation trajectory, ensuring a robust estimation of the thermodynamic properties [40, 41].

The binding energy (ΔG_bind) of each system, which consists of the complex, ligand, and receptor, was calculated using the following.

1 $$\Delta\:G_{bind} = G_{complex}-(G_{receptor} + G_{ligand})$$

This is further expressed as:

2 $$\Delta\:G_{bind} =\Delta\:G_{gas} + \Delta\:G_{sol}-{T}\Delta{S} $$

Where:

ΔG _gas_ = gas-phase interaction energy, calculated as:

3 $$\Delta\:G_{gas} = \Delta\:E_{VDWAALS} + \Delta\:E_{EEL}$$

ΔE_VDWAALS_ = van der Waals energy.

ΔE_EEL_ = Electrostatic (Coulombic) interaction energy.

ΔG_solv_ = solvation-free energy, calculated as:

4 $$\Delta\:G_{solv} = \Delta\:E_{GB} + \Delta\:E_{SURF}$$

ΔG_solv_ is the solvation-free energy consisting of polar and non-polar contributions.

ΔE_GB_: Polar solvation energy, computed using the Poisson-Boltzmann (PB) equation, which follows the Gibbs-Bogoliubov variational principle for approximating free energy in complex molecular systems [42].

ΔE_SURF_: The non-polar solvation energy is calculated based on the solvent-accessible surface area (SASA), utilizing a water probe radius of 1.4 Å. The ΔE_SURF_ or G_SURF_ term, which is proportional to the SASA, is determined as follows:

5 $$\Delta\:E_{SURF}= \gamma. {SASA} $$

γ (gamma) is an empirical parameter (often around 0.00542 kcal/mol/Å²) representing the surface tension of water. TΔS represents the entropic contribution, where the temperature is T, and the total entropy of the system is S. Although not directly computed in all MM/PBSA calculations, it is considered when comparing the relative affinity of binding between different protein-ligand systems.

### The density functional theory (DFT)

The molecular geometries of the two hit compounds were refined using Density Functional Theory (DFT), employing the B3LYP level of theory and 6-311 + + G(d, p) basis set in Gaussian 16 [43]. Vibrational frequency analysis verified that no imaginary frequencies were present in the optimized structures, confirming their stability. It also enabled the determination of frontier molecular orbitals (HOMO and LUMO), energy gap, and MEP.

## Results and discussions

### Molecular docking evaluation of xanthone derivatives as ACE2 inhibitors

A comprehensive molecular docking study was conducted on xanthone derivatives to evaluate their binding energy toward ACE2, a critical receptor involved in SARS-CoV-2 viral entry. This analysis included the prototype inhibitor MLN-4067, natural xanthone derivatives, and their tautomers, totaling 237 compounds docked against the ACE2 (PDB ID: 1R4L) receptor. The docking scores for the ACE2-ligand complexes ranged from -9.26 to -1.26 kcal/mol. These negative values indicate favorable and spontaneous binding of the xanthone derivatives to the ACE2 inhibitory site, suggesting their potential as effective ACE2 inhibitors. The binding free energies (in kcal/mol) provide insight into each compound’s relative binding energy, with more negative scores indicating stronger binding [44]. Interestingly, when compared to the prototype inhibitor MLN-4067 (binding energy of -6.9 kcal/mol), five xanthone derivatives, XAN71, XAN72, XAN7, XAN70, and XAN68, demonstrated superior binding affinities, making them top candidates for ACE2 inhibition. To further confirm the effectiveness of the applied method, we perform prime MM-PBSA calculation via Glide [45].

**Fig. 3:**
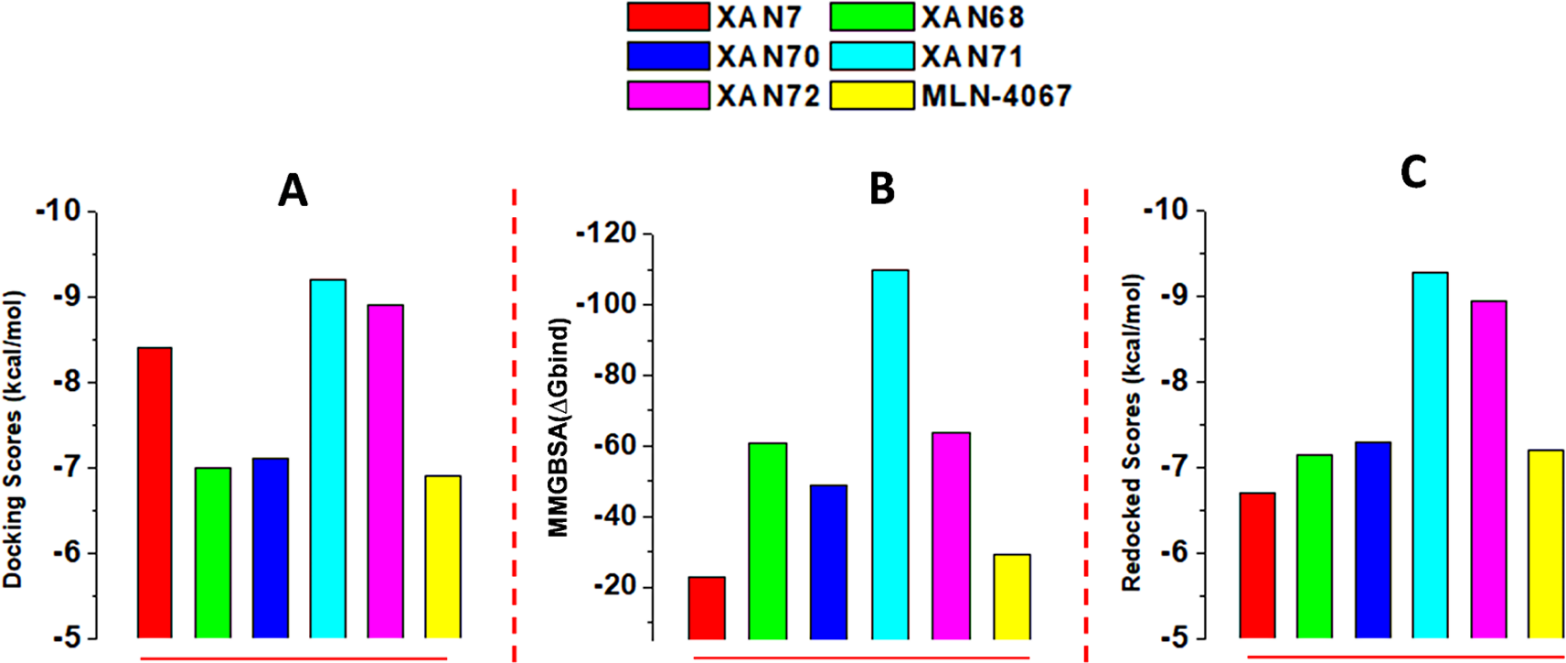
Molecular docking scores **(A)** Prime/MMGBSA binding energy (ΔGbind) **(B)** and molecular redocking scores **(C)** of the five lead compounds and the prototype inhibitor with ACE2

**Table 1:** Glide docking, prime MM/GBSA, and DFT optimization

SN	Compound	Glide docking score kcal/mol	Prime mmgbsa kcal/mol	Redocked score after DFT Optimization(kcal/mol)
1	XAN7	-8.41	-22.96	-6.70
2	XAN68	-7.01	-60.84	-7.15
3	XAN70	-7.12	-48.89	-7.32
4	XAN71	-9.26	-109.94	-9.28
5	XAN72	-8.93	-63.56	-8.95
6	MLN-4760	-6.91	-29.07	-7.20

As illustrated in Fig. 3; Table 1, the results from Glide docking reveal that XAN71 (-9.26 kcal/mol) and XAN72 (-8.93 kcal/mol) demonstrated the highest binding affinities compared to the reference inhibitor MLN-4760 (-6.91 kcal/mol). These values indicate that both xanthone derivatives exhibit strong interaction potential within the ACE2 active site, surpassing the prototype ligand’s ability to occupy and stabilize within the binding pocket. To further validate the binding stability, Prime MM/GBSA calculations were performed**(L**). This method integrates solvation effects and interaction energies, offering a more comprehensive evaluation of binding affinities. The results showed significantly lower (more favorable) binding energies for XAN71 (-109.94 kcal/mol) and XAN72 (-63.56 kcal/mol), underscoring their superior interaction stability compared to MLN-4760 (-29.07 kcal/mol). Interestingly, XAN71 exhibited the strongest binding energy among all tested compounds, highlighting its potential as a leading candidate for ACE2 inhibition. Following DFT optimization of the ligand geometries, redocking was conducted to refine and reassess the interaction energies. Post-DFT optimization, XAN71, and XAN72 retained their superior binding scores (-9.28 kcal/mol and − 8.95 kcal/mol, respectively). While XAN68 and the reference MLN-4067 also improved their scores, XAN7 exhibited an exception with the lowest score of 6.70 kcal/mol. The enhanced scores can be attributed to the optimized geometry, which likely improved spatial alignment and interactions with key residues within the binding pocket. The improved energy values demonstrate that DFT optimization enhances molecular conformations, resulting in better accommodation within the receptor’s active site and stronger binding interactions.

### Interaction analysis

The activity of enzymes is closely associated with a specific region within their structure known as the active site, typically shaped as a cavity or groove. Ligands or inhibitors interact with the active site of the target enzyme by forming connections with surface residues located within this cavity, particularly with specific residues that characterize the active site. In the case of the ACE2 protein, the binding cavity is characterized by key residues, including TYR510, LYS363, ASP368, PRO346, ARG273, THR371 and HIS345. The prototype inhibitor MLN-4067 interacts with these residues within the ACE2 binding site, stabilizing its binding through van der Waals forces, hydrogen bonds, hydrophobic, and other key interactions that influence its inhibitory activity [25, 39]. Ligands interacting with this receptor are expected to develop a variety of interactions with some of these amino acids.

**Fig. 4:**
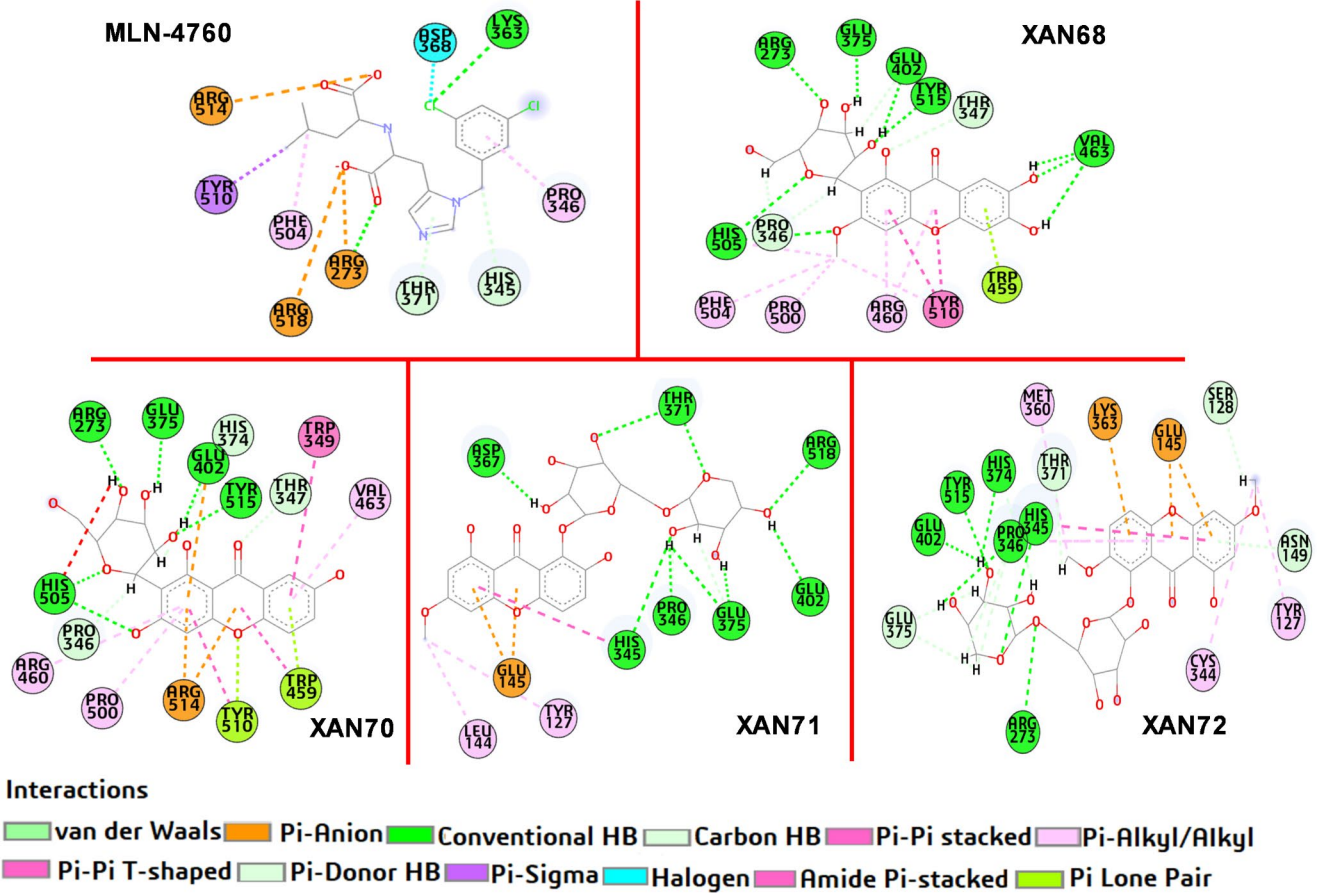
2D representation of the interaction of top 4 ranked ligands plus the reference with ACE2

A detailed examination of the binding interactions of the top four xanthone derivatives with ACE2 (Fig. 4) reveals a complex network of stabilizing interactions within the ACE2 active site. These interactions include conventional and unconventional hydrogen bonds, hydrophobic contacts, π-π stacking, and electrostatic interactions, all contributing to enhanced binding energy and stability. The lead compounds exhibit key pharmacophore features that facilitate specific interactions with ACE2. For instance, XAN71 and XAN72 possess hydroxyl and carbonyl groups that enable strong hydrogen bonding with polar residues, while their methoxy groups contribute to hydrophobic interactions with residues such as TYR127 and LEU144. Notably, XAN72 forms unconventional C-H bonds with THR371, and both compounds support π-π interactions through their aromatic rings. Hydrophobic moieties further enhance van der Waals interactions, improving ligand fit within the ACE2 binding site. Among these compounds, XAN71 demonstrates the highest stability due to its formation of 11 conventional and 2 unconventional hydrogen bonds with critical residues, including THR371, ARG518, GLU402, GLU375, PRO346, and HIS345. Importantly, while XAN71 and XAN72 show favorable interactions with ACE2, they do not share common amino acid interactions with the reference inhibitor MLN-4067. This lack of overlap may provide a beneficial specificity for these xanthone derivatives as potential inhibitors against SARS-CoV-2.

Similarly, XAN72 demonstrates strong stabilization through 7 conventional and 8 unconventional hydrogen bonds with residues such as ARG273, HIS345, and TYR515. Both ligands also establish electrostatic interactions, with XAN71 forming π-anion contacts with GLU145 and XAN72 establishing interactions with LYS363 and GLU145. These π-π stacking interactions, particularly with TYR510 and HIS345, stabilize ligand orientation, enhancing fit within the binding pocket. The top 4 xanthone derivatives, including XAN68 and XAN70, demonstrate an improved interaction profile over MLN-4067. Their strong networks of hydrogen bonds, along with hydrophobic interactions, π-π stacking, and van der Waals forces, contribute to a more stable and energetically favorable ligand-protein complex. These interactions highlight the enhanced binding potential of xanthone derivatives as ACE2 inhibitors, offering greater stability and efficacy than the prototype compound, MLN-4067.

However, despite the favorable outcomes from the initial docking and re-docking processes, further analysis was essential to prove the stability and reliability of the interactions between the top 4 xanthone derivatives and the ACE2 receptor. Docking scores, while valuable, provide a static snapshot of the binding energy and do not account for the dynamic nature of molecular interactions within a biological environment [46]. Therefore, the top candidate compounds were subjected to (MD) simulations to obtain a more comprehensive understanding of the stability and effectiveness of the ligand-protein interactions.

### Molecular dynamic simulation

MD simulations enable observing the behavior of ligand-protein complexes over time, replicating the fluctuating conditions present in a biological environment [47, 48]. This step provides crucial information on binding stability and interaction dynamics. By running MD simulations, we can assess the stability of the ligand within the ACE2 binding pocket. This helps to understand if the ligand maintains a stable binding pose over time or if it dissociates, which is critical for determining the practical efficacy of the compound [49]. The simulation also allows tracking of specific ligand-protein interactions, including hydrogen bonds, van der Waals contacts, and hydrophobic interactions. These dynamic interactions reveal how consistently the ligand engages with key amino acids in the binding pocket site, offering insights into the robustness of the binding energy [50]. After the MD runs of 200 ns were completed, post-MD were calculated and analyzed accordingly.

### Post MD analysis

The binding of an inhibitor to a specific biological target typically induces structural and conformational changes that can significantly influence the target’s biological function [51]. To investigate these structural modifications and their potential impact on the protein’s activity, we evaluated the Root Mean Square Deviation (RMSD), Radius of Gyration (RoG), and Root Mean Square Fluctuation (RMSF) of the alpha carbon (Cα) atoms for both the unbound protein and the inhibitor-bound complex. These parameters were monitored throughout a 200 ns molecular dynamics (MD) simulation to provide insights into the dynamic behavior over time. Additionally, we performed Molecular Mechanics/Poisson–Boltzmann Surface Area (MM/PBSA) calculations [52, 53] as part of the post-MD analysis to assess the binding affinities of the potential inhibitors. This approach allows for a comprehensive evaluation of the free energy changes associated with ligand binding, further elucidating how the observed structural alterations correlate with binding stability and affinity changes.

### Binding energy calculation using the MM-PBSA method

**Table 2:** Binding energy components of the molecular complexes in Kcal/mol

Complexes	∆E_vdWaals_	∆E_elec_	∆E_GB_	∆E_surf_	∆G_gas_	∆G_Solv_	∆G_bind_
MLN-4067	-45.01 ± 3.83	-528.86 ± 13.16	519.22 ± 10.15	-6.69 ± 0.21	-573.86 ± 13.01	512.52 ± 10.05	-61.33 ± 4.62
XAN68	-47.96 ± 5.06	-71.44 ± 18.34	-78.17 ± 11.71	-6.22 ± 0.20	-116.41 ± 16.93	71.95 ± 11.62	-44.46 ± 8.86
XAN70	-43.96 ± 4.04	-50.99 ± 3.92	58.55 ± 7.34	-5.42 ± 0.33	-90.08 ± 13.90	53.13 ± 7.14	-36.95 ± 8.45
XAN71	-76.65 ± 3.76	-72.71 ± 7.85	88.10 ± 6.30	-9.71 ± 0.15	-149.36 ± 7.97	78.39 ± 6.29	-70.97 ± 4.50
XAN72	-75.14 ± 4.21	-91.79 ± 11.25	106.49 ± 8.09	-9.41 ± 0.37	-166.93 ± 10.59	97.08 ± 8.06	-69.85 ± 5.28

**∆**E_vdW_ - van der Waals energy, **∆**E_ele_ - electrostatic energy, ΔE_GB −_ polar solvation energy, ΔE_SURF −_ non-polar solvation energy **∆**G_sol_ - solvation free energy, **∆**E_gas −_ gas-phase free energy, ∆G_bind_ - total binding free energy

The MM/PBSA method, as described by Hollingsworth SA [49], was used to estimate the binding energy (ΔG_bind_) between a ligand and a receptor [51]. Following 200 ns MD simulations, we assessed the binding energy of the prototype ligand MLN-4067 at the active sites of ACE2. Table 2 presents the binding energy components of ACE2 complexes with MLN-4067 and xanthone derivatives XAN68, XAN70, XAN71, and XAN72. These values provide insight into each compound’s binding energy and interaction stability [49]. The prototype inhibitor MLN-4067 shows a substantial ΔG_gas (-573.86 kcal/mol), driven by strong electrostatic interactions. However, its binding energy ΔG_bind is moderate (-61.33 kcal/mol) due to a high solvation penalty (+ 512.52 kcal/mol), which offsets much of its gas-phase affinity. Among the xanthone derivatives, XAN71 exhibits the strongest binding energy with ΔG_bind = -70.97 kcal/mol, attributed to favorable van der Waals and electrostatic interactions with values of -76.65 and  -72.71 kcal/mol, respectively, combined with a moderate solvation penalty (+ 78.39 kcal/mol). XAN72 follows closely with ΔG_bind = -69.85 kcal/mol, also driven by strong gas-phase interactions. In contrast, XAN68 and XAN70 show lower binding affinities (ΔG_bind = -44.46 kcal/mol and -36.95 kcal/mol, respectively), largely as a result of less favorable gas-phase interactions and relatively high solvation penalties. These findings align with our earlier docking scores, where XAN71 and XAN72 displayed higher affinities than XAN68 and XAN70. The MM/PBSA calculations further confirm that XAN71 and XAN72 exhibit the highest binding stability, with XAN71 showing the strongest overall binding energy, positioning it as a promising candidate for ACE2 inhibition. The combination of balanced van der Waals, electrostatic contributions, and moderate solvation penalties enhances their binding efficacy compared to the prototype inhibitor MLN-4067.

### Protein and Ligand RMSD

**Fig. 5:**
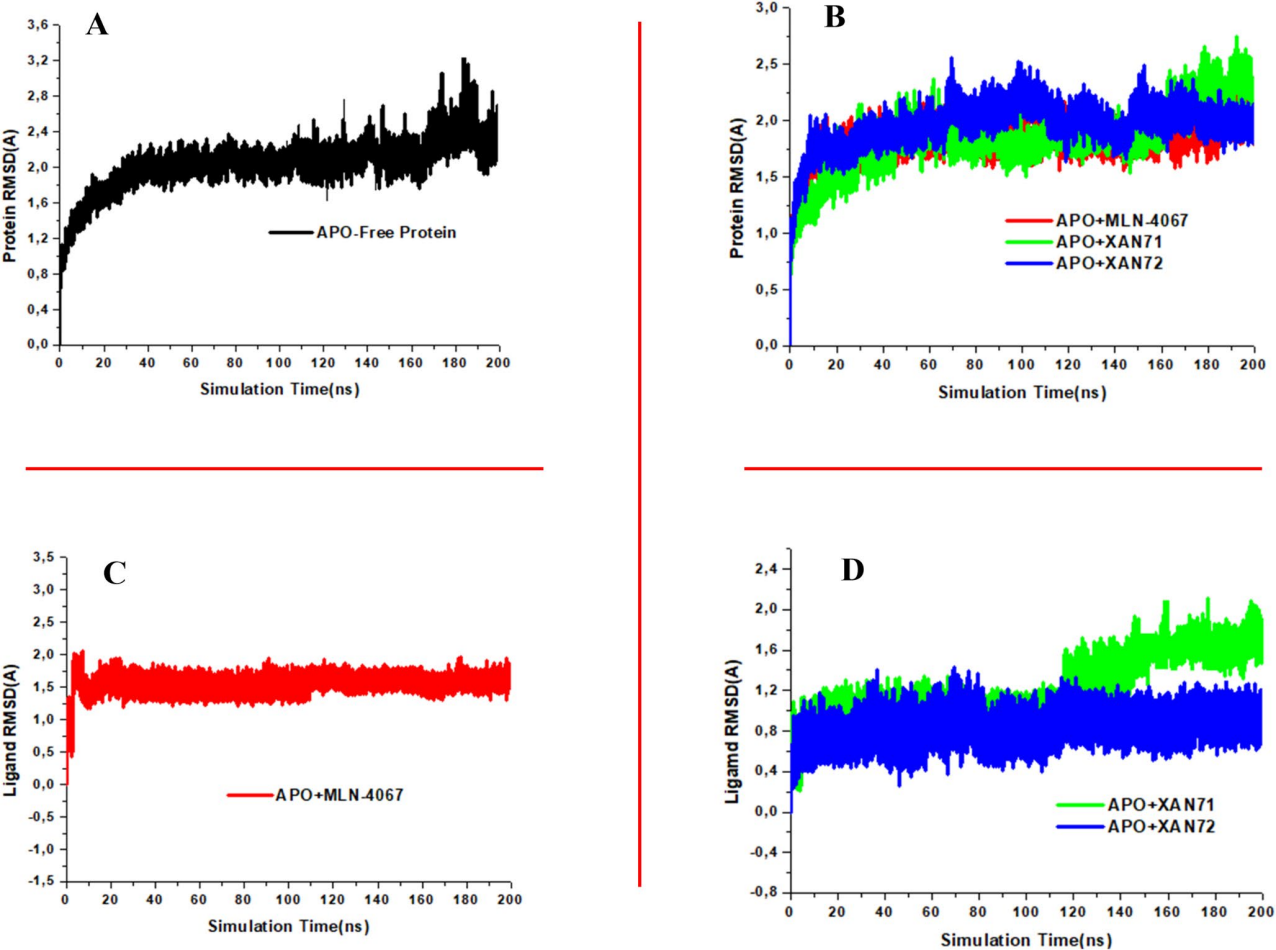
Protein RMSD (**A**) APO-Free Protein (**B**) Bound Systems and Ligand RMSD (**C**) APO-MLN-4067 (**D**) Bound Systems plots against ACE2

The behavior of the protein backbone and ligands within the ACE2 receptor’s binding pocket was assessed using RMSD trajectory analysis. The structural organization of a protein is a key determinant of its biological function, and any significant structural alterations may positively or negatively impact its activity [54]. Protein RMSD provides insight into the structural stability of the unbound (apo) system compared to its complex forms with XAN71 and XAN72, revealing the degree of deviation in the protein’s Cα atoms. Low, stable RMSD values suggest maintained structural integrity, whereas higher or fluctuating RMSD values indicate decreased stability and potential conformational changes [55]. In Fig. 5A, the RMSD of the apo ACE2 protein shows an initial increase, reaching approximately 1.8 Å, before stabilizing around 20 ns. It maintains a relaxed conformation with fluctuations between 2.0 Å and 3.0 Å throughout the simulation. A slight deviation is observed between 170 ns and 180 ns, after which the system regains stability and remains consistent for the remainder of the 200 ns simulation. This reasonably stable behavior validates the molecular dynamics (MD) protocol used for our protein-ligand complex simulations. In Fig. 5B, both complexes with XAN71 and XAN72 demonstrate lower average RMSD values than the free protein, with XAN71 showing an average RMSD of 1.84 Å and XAN72 at 1.96 Å. These results indicate that both complexes are stable, with XAN72’s RMSD resembling the free proteins, suggesting comparable structural stability. Throughout the simulation, all systems displayed average RMSD values below 2 Å, indicating stable interactions between XAN71 and XAN72 with ACE2’s active site residues. For XAN71, the RMSD exhibited an initial increase up to approximately 20 ns, followed by minor fluctuations around 25 ns. The system reached convergence by 30 ns and maintained a relaxed and stable conformation for roughly 70% of the simulation. However, a slight rise in RMSD was observed near 170 ns, after which the system regained stability and remained steady for the remainder of the 200 ns simulation. Similarly, XAN72 exhibited an initial increase up to 10 ns, followed by minor fluctuations around 70 ns and a slight drop near 100 ns, maintaining stability for most of the simulation. The average RMSD for the prototype inhibitor MLN-4067 Fig. 5C. was recorded at 1.83 Å, showing a slight rise up to 10 ns before remaining stable throughout the simulation. While both XAN71 and XAN72 demonstrated superior stability compared to MLN-4067, their distinct binding interactions may offer unique advantages as potential inhibitors. The absence of common amino acid interactions between XAN71, XAN72, and MLN-4067 suggests that these xanthone derivatives could provide enhanced specificity in targeting ACE2 without competing directly with established inhibitors. Overall, the consistent RMSD values throughout most of the simulation suggest stable ligand-protein interactions for both xanthone derivatives, supporting their compatibility within the ACE2 binding pocket and highlighting their potential as effective therapeutic agents against SARS-CoV-2.

Ligand RMSD was analyzed using trajectory data to assess the behavior of ligands within the ACE2 binding pocket [47]. RMSD values provide insights into the stability of each ligand’s binding mode; high RMSD values indicate significant movement and less stable binding, while low RMSD values suggest consistent interactions with key residues in the binding pocket [56]. From Fig. 5C, the prototype inhibitor MLN-4067 displayed an average RMSD of 1.54 Å, with slight fluctuations observed up to 10 ns before relaxation for the remainder of the simulation. In contrast, XAN71 maintained a low and stable RMSD, averaging 1.20 Å between 2 ns and 120 ns, with only a slight increase observed before stabilizing for the remainder of the simulation. This consistency suggests strong and stable interactions with key residues in the active site, with no evidence of dissociation throughout the simulation. XAN72 demonstrated an even lower average RMSD of 0.85 Å, achieving equilibrium from the start and maintaining it throughout the 200 ns simulation. The low average RMSD values for XAN71 (1.20 Å) and XAN72 (0.85 Å) highlight their stability and consistent binding orientation within the ACE2 binding pocket. RMSD values below 1 Å indicate minimal positional drift [57], suggesting that both ligands establish stable, favorable interactions with key residues in the binding site. This stable behavior reinforces the suitability of XAN71 and XAN72 as potential ACE2 inhibitors, supporting robust ligand-protein interactions. When comparing these compounds to MLN-4067, XAN71 and XAN72 exhibit superior stability, as indicated by their lower RMSD values. The enhanced stability of XAN71 and XAN72 suggests that they may provide more effective inhibition of ACE2 than MLN-4067, potentially leading to better therapeutic outcomes. Collectively, these results validate the robustness of the XAN71-ACE2 and XAN72-ACE2 complexes, underscoring their promise as effective candidates in targeting SARS-CoV-2.

### Protein RSMF and Protein RoG

**Fig. 6:**
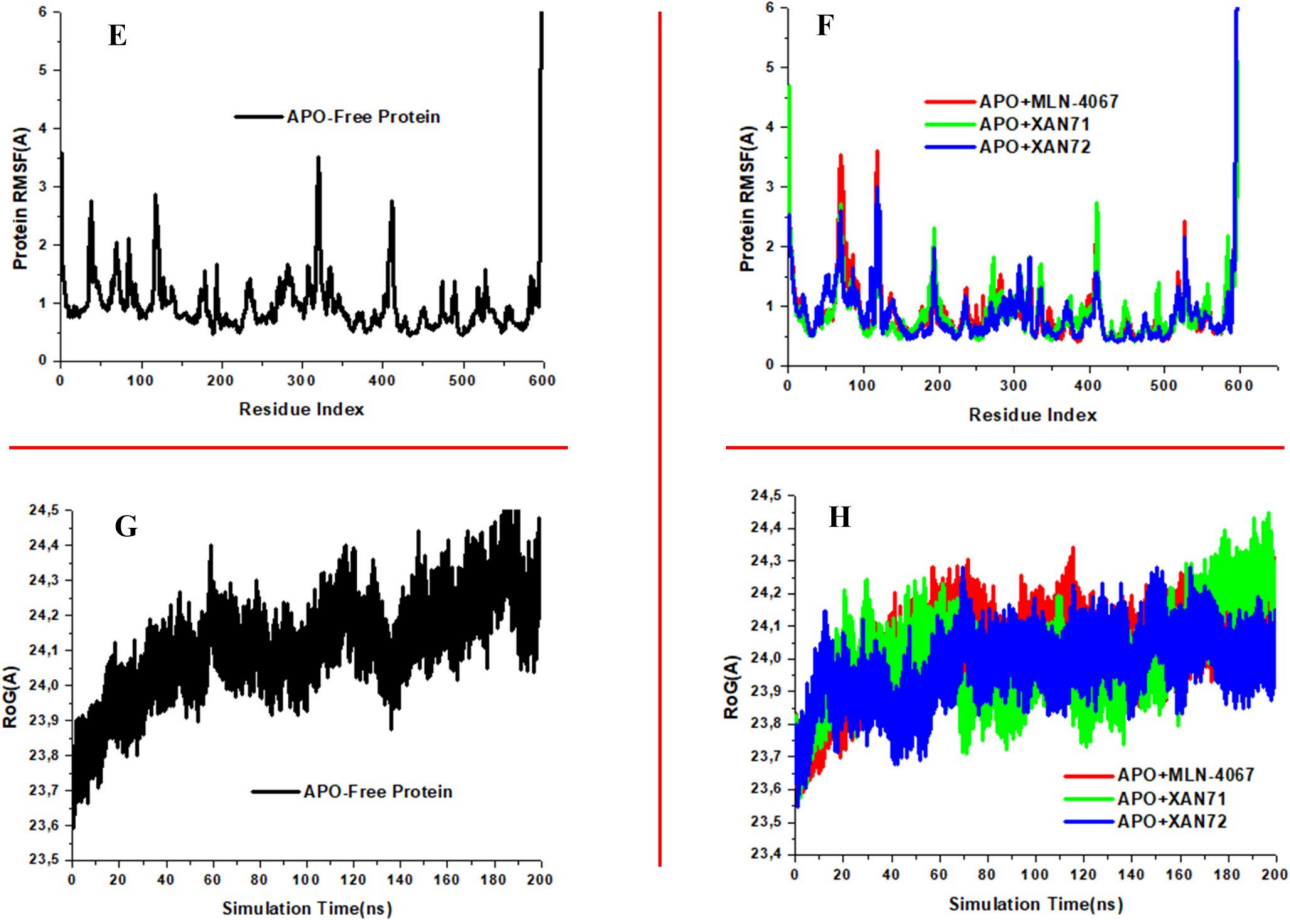
Protein RMSF (**E**) APO-Free Protein (**F**) Bound Systems Protein RoG (**G**) APO-Free Protein (**H**) APO-MLN-4067 and Bound Systems plots against ACE2

The stability of protein-ligand complexes is significantly influenced by individual amino acid residues [58]. Analyzing the Root Mean Square Fluctuation (RMSF) trajectory provides valuable insights into the flexibility of various regions within a protein’s structure during molecular dynamics (MD) simulations, particularly in response to ligand binding. RMSF analysis highlights the role of amino acids in stabilizing the protein-ligand complex by identifying areas with varying levels of flexibility. Typically, high RMSF values indicate flexible or loop regions, whereas low RMSF values are associated with stable or rigid areas, often corresponding to secondary structural elements [59]. In this study, we calculated and plotted the RMSF values for the free ACE2 protein and its complexes with XAN71 and XAN72, as shown in Fig. 6E and F. The results reveal a notable reduction in ACE2 flexibility upon binding with XAN71 and XAN72, with both complexes exhibiting an average RMSF of 0.87 Å, compared to 0.89 Å for the prototype inhibitor MLN-4067 and 0.96 Å for the free protein. This decrease in RMSF indicates enhanced structural stability of ACE2 when bound to these ligands, suggesting that XAN71 and XAN72 effectively stabilize the ACE2 active site. The RMSF analysis also illustrates fluctuations across the protein’s residue indices, highlighting conformational changes that could impact its function. Notably, the free ACE2 protein exhibited greater fluctuations in several residues compared to the XAN71 and XAN72 complexes, particularly in regions outside the active site domain, such as between residues 350 and 400. This observation indicates that ACE2 becomes less flexible upon ligand binding. The lower RMSF values observed in the ligand-bound systems suggest enhanced structural compactness, which may influence ACE2’s functional integrity by potentially preventing viral entry into host cells [46, 60]. Additionally, regions in the apo (free) ACE2 structure displayed fluctuations exceeding 3.0 Å, especially around residues 0–10 and 320–325, indicating increased atomic movement in these domains. In contrast, the XAN71 and XAN72 complexes showed fewer fluctuations, reflecting less conformational variability in their ligand-bound states. Overall, the reduced RMSF values for the XAN71-ACE2 and XAN72-ACE2 complexes imply stabilized interactions, decreased conformational flexibility, and a more compact protein structure—all factors that support the inhibitory potential of XAN71 and XAN72 against ACE2, thereby limiting viral entry [57].

The rigidity of protein-ligand complexes can be evaluated using the Radius of Gyration (RoG) parameter derived from molecular dynamics (MD) simulation trajectories. The RoG reflects the distribution of a protein’s mass relative to its center of mass, providing valuable insights into its overall compactness [61]. Lower RoG values typically indicate a more compact and stable structure, while higher RoG values may suggest unfolding or structural loosening [62]. Figure 6G and H display the RoG versus time plots for the systems analyzed. The average RoG values for the XAN71-bound (23.98 Å) and XAN72-bound (23.97 Å) complexes, as well as for the prototype inhibitor MLN-4067 (24.02 Å), were slightly lower than that of the unbound protein (24.11 Å), suggesting enhanced compactness upon ligand binding. In Fig. 6G, fluctuations in the RoG of the unbound protein are evident around 60 ns, 90 ns, 110 ns, and 135 ns, indicating periods of reduced compactness. Conversely, the XAN71 and XAN72 complexes exhibited an initial rise in RoG, stabilizing around 15 ns, with minor fluctuations around 40 ns before achieving stable compactness until the end of the simulation. Overall, these findings indicate that the compactness of the protein structure in the XAN71 and XAN72-bound systems remains stable and is not adversely affected by ligand binding. This stability further supports the structural integrity and rigidity of the complexes throughout the simulation period. The consistently lower RoG values for both ligand-bound systems compared to the free protein underscore their potential to maintain a more compact conformation, which is beneficial for effective inhibition of ACE2 and may enhance resistance to conformational changes that could facilitate viral entry.

### Per-residue energy decomposition (PRED) analysis

**Fig. 7:**
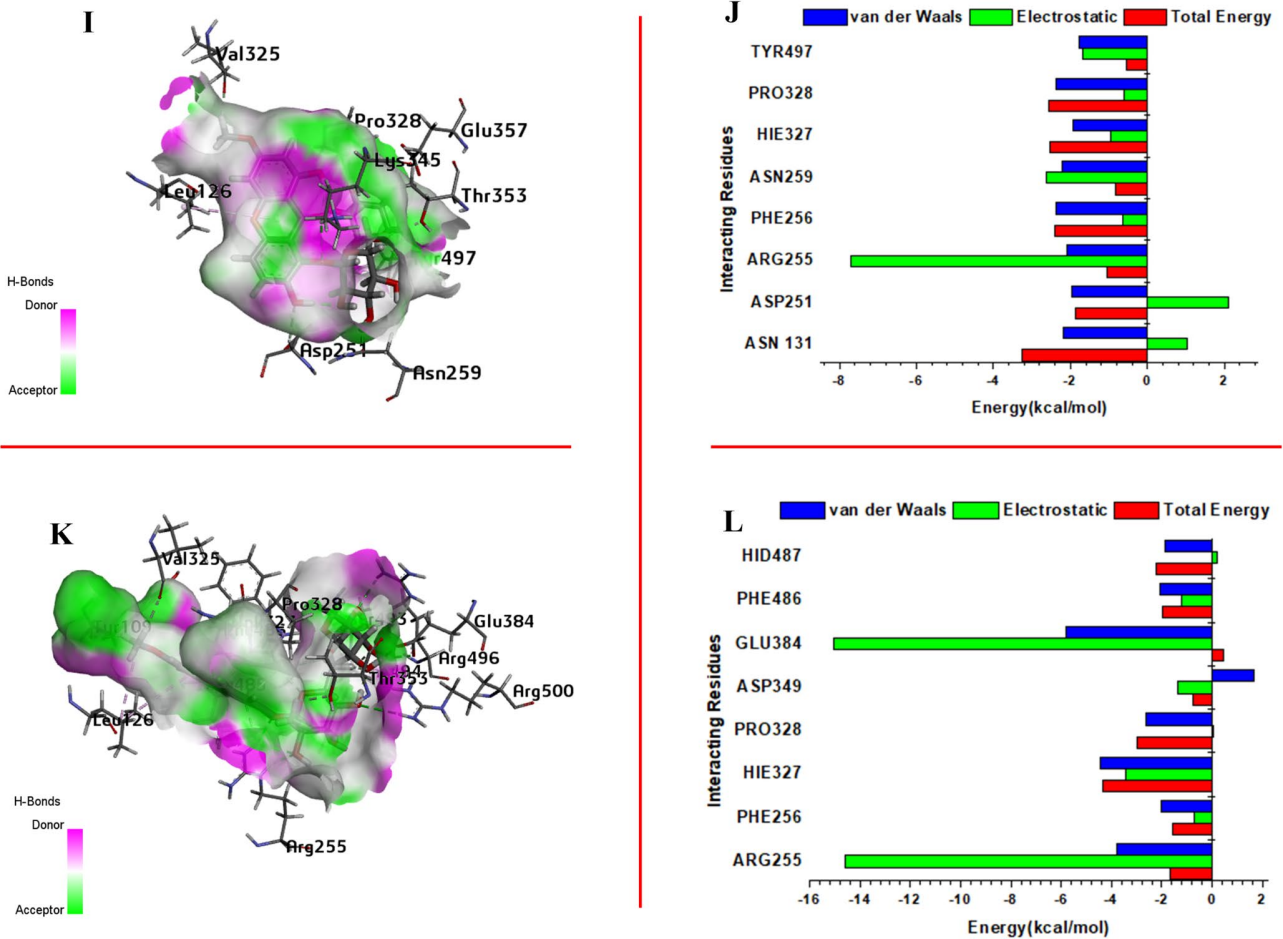
3D structure of the XAN71-ACE2 complex and XAN72-ACE2 complex (**I**, **K**) Per-Residue Energy Decomposition (PRED) of XAN71 and XAN72 with ACE2 (**J**, **L**)

A per-residue energy decomposition data (PRED) was generated as part of the MM/PBSA calculations to comprehend how individual residues contribute to ligand binding. This data highlights the essential residues that play a significant role in the binding energy of XAN71 and XAN72 to ACE2 protein. The eight residues with the highest energy contributions for each ligand complex are displayed in Fig. 7J and L, alongside their respective 3D interaction structures, highlighting hydrogen bonding regions.

For the XAN71-ACE2 complex (Fig. 7J), residues such as ASN131 and ARG255 showed significant contributions to the binding, with total energy values of -2.19 kcal/mol and -2.08 kcal/mol, respectively. These energies arise from van der Waals (vdW) and electrostatic interactions. Additionally, PHE256 and PRO328 exhibited notable vdW contributions, emphasizing their role in stabilizing hydrophobic interactions within the binding pocket. The overall binding free energy of XAN71 was primarily influenced by vdW forces, resulting in a highly favorable ΔGbind of -70.97 kcal/mol. In the case of the XAN72-ACE2 complex (Fig. 7L), ARG255 and GLU384 were identified as the most significant contributors, with total energy values of -3.78 kcal/mol and -5.83 kcal/mol, respectively. The substantial electrostatic contribution from GLU384 (-15.05 kcal/mol) highlights the critical role of ionic and polar interactions in the binding of XAN72.

Furthermore, residues HIE327 and PHE486 contributed significantly through both vdW and electrostatic interactions, reinforcing the structural stability of the complex. The overall binding free energy for XAN72 was measured at -69.85 kcal/mol, slightly lower than that of XAN71, indicating comparable binding affinities. The residue-level decomposition analysis illustrates the interplay between vdW and electrostatic forces in stabilizing these ligand-receptor complexes. Molecular dynamics (MD) simulations confirmed enhanced binding energies for both XAN71 and XAN72, validating the efficacy of the MM-PBSA method in quantifying interaction contributions. The predominance of van der Waals interactions suggests that the multi-ringed structure of these xanthone derivatives is particularly well-suited for engaging with the non-polar regions of the ACE2 active site, thereby enhancing ligand binding and stability.

### Density functional theory studies

Following the (MD) analysis, a DFT study assessed the chemical reactivity and electrostatic properties of XAN71 and XAN72. This investigation provides additional insights into their binding behavior and potential interactions with ACE2. Among the various reactivity descriptors, Molecular electrostatic potentials (MEPs) are frequently utilized to predict sites for electrophilic and nucleophilic attacks and enhance the understanding of biological recognition and hydrogen-bonding interactions. The MEP maps are color-coded from red to blue to represent different electrostatic potentials: deep red indicates electron-rich regions prone to electrophilic attack, while blue areas signify electron-deficient regions favoring nucleophilic attack. Green regions are near-neutral, and yellow areas denote regions with lower electron density.

**Fig. 8:**
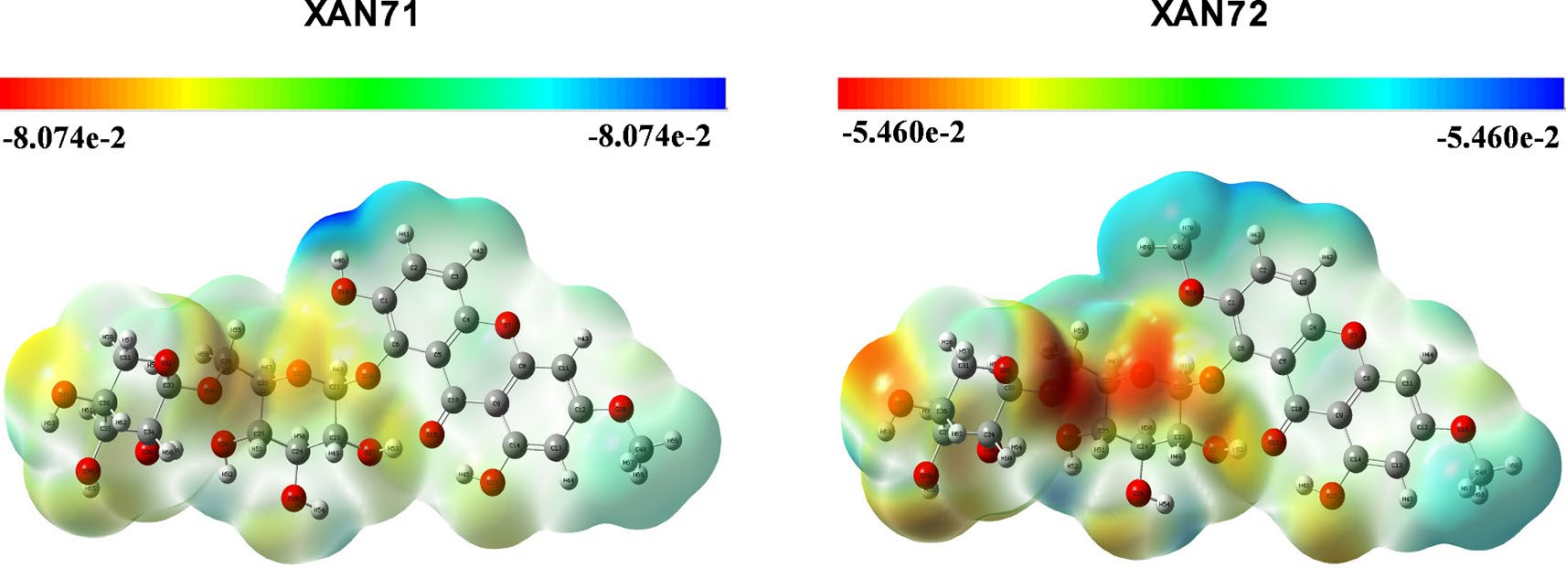
Molecular electrostatic potentials of the XAN71 and XAN72

Figure 8 presents the MEP maps for XAN71 and XAN72, which share similar regions of light blue on the xanthone ring, indicating electron-deficient sites. Slightly red regions are observed around the pyran rings, particularly near the 6-hydroxy groups, suggesting sites of potential electrophilicity. Notably, XAN71 has a deep blue area around the hydroxyl group at position 8 on the xanthone ring, indicating a site of nucleophilicity favorable for hydrogen bonding interactions. In contrast, XAN72 differs only in having a methoxy group at the same position, resulting in slightly altered electrostatic potential distribution. The MEP maps align with the hydrogen-bonding and hydrophobic interactions observed in the MD analysis, particularly around the hydroxyl, methoxy, and aromatic groups. These features enhance polar interactions and hydrogen bonding, reinforcing the stability of the XAN71 and XAN72 complexes with ACE2.

### The HOMO-LUMO studies

**Fig. 9:**
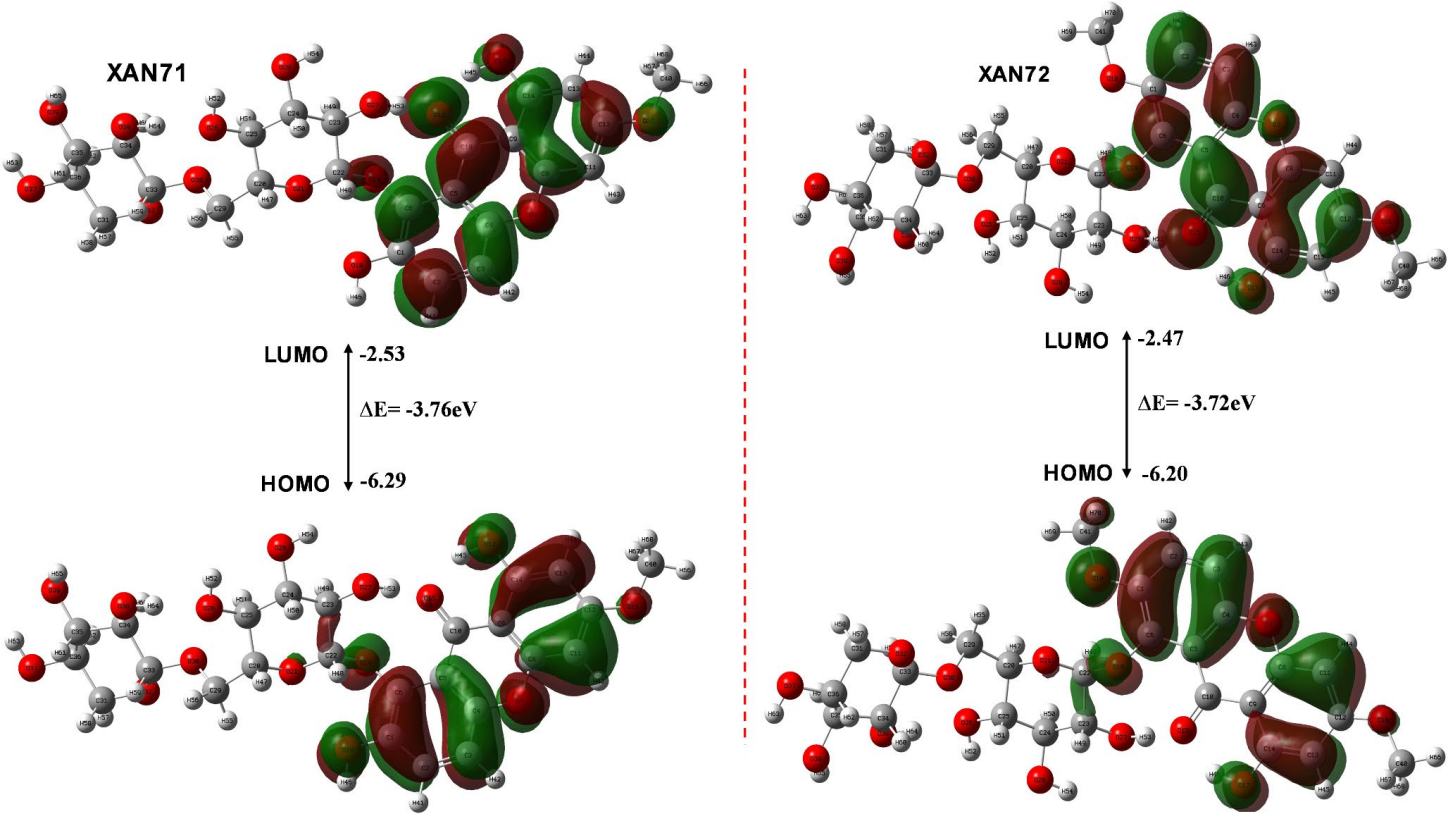
Frontier molecular orbitals LUMO (top) and HOMO (bottom)

The HOMO-LUMO energy gap (ΔE) offers valuable insights into the chemical reactivity and stability of XAN71 and XAN72, as shown in Table 3; Fig. 9. By examining the energies of the HOMO and LUMO, we can better understand the potential binding interactions of each compound with the ACE2 receptor. For XAN71, the LUMO energy is measured at -0.093 a.u. (or -2.532 eV), while XAN72 has a slightly lower LUMO energy of -0.090 a.u. (or -2.474 eV). The higher LUMO energy of XAN71 suggests that it may be marginally less reactive towards electron-rich regions than XAN72, as lower LUMO energies typically indicate easier access for electron-donating interactions.

**Table 3:** HOMO and LUMO Energies and their gap ΔE_Gap_

Parameter	XAN71_au_	XAN71_eV_	XAN72_au_	XAN72_eV_
^***ε***^ **LUMO**	-0.093	-2.532	-0.090	-2.474
^***ε***^ **HOMO**	-0.231	-6.299	-0.227	-6.203
**∆E**_**Gap**_	0.138	3.767	0.137	3.729

au = atomic unit, eV = electron volts, 1au = 27.2116 eV

As illustrated in Fig. 9, the LUMO and HOMO regions are highlighted in green and brown, respectively. The combination of these colors delineates the LUMO, while the same applies to the HOMO. Notably, the spatial arrangement of the green and brown orbitals for the LUMO differs from that of the HOMO, indicating potential interaction sites where electron density could facilitate bonding with electron donors. The HOMO energy for XAN71 is higher at -0.231 a.u. (or -6.299 eV), compared to -0.227 a.u. (or -6.203 eV) for XAN72. This slightly elevated HOMO energy for XAN71 suggests it may act as a stronger electron donor, potentially enhancing interactions with electron-deficient regions in the ACE2 binding site. In Fig. 9, the HOMO regions are depicted in green and brown, showcasing areas with increased electron density that may engage in interactions with electron-deficient sites within the receptor’s binding domain. Both compounds exhibit comparable HOMO-LUMO energy gaps, with XAN71 at 0.138 a.u. (or 3.767 eV) and XAN72 at 0.137 a.u. (or 3.729 eV).

The marginally smaller energy gap of XAN72 may indicate slightly higher reactivity than XAN71, potentially improving its adaptability in binding interactions. The energy gap illustrated in Fig. 9 reflects the relative stability and reactivity of the compounds; a smaller gap generally correlates with increased reactivity. The spatial distribution of HOMO and LUMO orbitals across each ligand emphasizes regions likely to interact with ACE2. For both XAN71 and XAN72, significant electron density is observed around the xanthone ring and functional groups such as hydroxyl and methoxy, which could facilitate hydrogen bonding or electrostatic interactions. This distribution aligns with the calculated reactivity, supporting stable binding interactions with ACE2 for both compounds while suggesting that XAN72 may have a slight advantage in reactivity due to its smaller energy gap. Conversely, the higher HOMO and LUMO energies of XAN71 may contribute to its overall stability, resulting in distinct binding characteristics that could enhance its effectiveness as an inhibitor. All these electronic properties suggest that these lead compounds are well-positioned to engage in favorable interactions with ACE2, potentially impeding viral entry into host cells.

## Conclusion

In this study, we identified XAN71 and XAN72, natural xanthone derivatives, as promising inhibitors of the ACE2 receptor, a critical target for SARS-CoV-2 entry into human cells. Molecular docking and molecular dynamics (MD) simulations demonstrated that both compounds formed stable, high-affinity interactions within the ACE2 binding domain, primarily through hydrogen bonding and hydrophobic interactions with key residues. Additionally, Molecular Electrostatic Potential (MEP) analysis highlighted electron-dense regions corresponding to potential binding sites, reinforcing the structural compatibility of these derivatives with ACE2.

While these findings provide valuable insights, the study has limitations that must be acknowledged. The conclusions are drawn solely from computational analyses, and no experimental validation has been conducted to confirm the predicted inhibitory effects. Furthermore, the pharmacokinetic, toxicity, and safety profiles of XAN71 and XAN72 should be exploited due to their importance in advancing these compounds as therapeutic candidates.

Future research should prioritize experimental validation in cellular and in vivo models to substantiate the computational predictions. Investigations into these compounds’ pharmacokinetics, toxicity, and off-target interactions are essential to establish their safety and therapeutic potential. Structural optimization may enhance their binding affinity, selectivity, and drug-like properties. Moreover, exploring their activity against emerging SARS-CoV-2 variants and other ACE2-utilizing viruses could expand their antiviral applications.

By integrating experimental validation with computational advancements, including machine learning-based approaches, the development of xanthone-based ACE2 inhibitors can be accelerated. These efforts will enhance the therapeutic potential of XAN71 and XAN72 and contribute to the preparedness for current and future pandemics, offering innovative solutions for antiviral drug discovery.

## Electronic supplementary material

Below is the link to the electronic supplementary material.

Supplementary Material 1

## Data Availability

No datasets were generated or analysed during the current study.
