# Supplementary material for: In silico exploration of natural xanthone derivatives as potential inhibitors of severe acute respiratory syndrome coronavirus 2 (SARS-CoV-2) replication and cellular entry
Source: J Comput Aided Mol Des. 2025 Feb 17;39(1):7. doi: 10.1007/s10822-025-00585-5 (PMC11832685; doi:10.1007/s10822-025-00585-5)
Supplement: Supplementary file 1 — Supplementary Material 1 [file 10822_2025_585_MOESM1_ESM.docx]

**SUPPLEMENTARY MATERIAL**

**In Silico Exploration of Natural Xanthone Derivatives as Potential Inhibitors of Severe Acute Respiratory Syndrome Coronavirus 2 (SARS-CoV-2) Replication and Cellular Entry.**

Vincent A. Obakachi^1*^, Vaderament-A. Nchiozem-Ngnitedem^2^ , Krishna K. Govender^1*^, Penny P. Govender^1^

^1^Department of Chemical Sciences, University of Johannesburg, Doornfontein Campus, P.O. Box 17011, Johannesburg 2028, South Africa.

^2^Institut für Chemie, Universität Potsdam, Karl-Liebknecht-Str. 24-25, D-14476 Potsdam-Golm, Germany.

**Table S1**. Anti-viral xanthone derivatives from natural sources

| **Compounds** | **Sources** | **Activity** | **References** |
| --- | --- | --- | --- |
| **Simple Xanthones** |  |  |  |
| 3,8-Dihydroxy-6-methyl-9-oxo-9*H*-xanthene-1-carboxylate (**1**) | *Diaporthe sp.* | Inhibits A/Puerto Rico/8/34 H274Y (H1N1) (IC_50_: 9.4 µM), A/FM-1/1/47 (H1N1)) (IC_50_: 4.8 µM), and A/Aichi/2/68 (H3N2)) (IC_50_: 5.1 µM) | [1] |
| 1,7-Dihydroxyxanthone (**2**) | *Garcinia oblongifolia* | Anti-EV71 (IC_50_: 12.2 µM) | [2] |
|  | *Polygala karensium* | Inhibits H1N1 (IC_50_: 23.5 µg/mL), H9N2 (IC_50_: 22.5 µg/mL), H1N1 (WT) (IC_50_: 11.5 µg/mL), H1N1 (H274Y) (IC_50_: 13.0 µg/mL) | [3] |
| Oliganthin E (**3**) | *Garcinia oligantha* | Anti-TMV (IC_50_: 125.2 µM) | [4] |
| Oliganthin F (**4**) | *Garcinia oligantha* | Anti-TMV (IC_50_: 109.5µM) | [4] |
| Oliganthin G (**5**) | *Garcinia oligantha* | Anti-TMV (IC_50_: 200 µM) | [4] |
| (*S*)-1,8-Dihydroxy-4-(1-hydroxy-3-oxobutyl)-3- methoxy-9H-xanthen-9-one (**6**) | *Garcinia oligantha* | Anti-TMV (15.2 % inhibitory at 20 μM) | [5] |
| Paucinervin E (**7**) | *Garcinia paucinervis* | Anti-TMV (IC_50_: 21.4 µM) | [6] |
| Paucinervin F (**8**) | *Garcinia paucinervis* | Anti-TMV (IC_50_: 42.8 µM) | [6] |
| Paucinervin G (**9**) | *Garcinia paucinervis* | Anti-TMV (IC_50_: 53.6 µM) | [6] |
| 1,3-Dihydroxyxanthone (**10**) | *Polygala karensium* | Inhibits H1N1 (IC_50_: 23.3 µg/mL), H9N2 (IC_50_: 15.5 µg/mL), H1N1 (WT) (IC_50_: 11.2 µg/mL), H1N1 (H274Y) (IC_50_: 7.7 µg/mL) | [3] |
| 4-Methoxy-2,3-methylenedioxyxanthone (**11**) | *Polygala karensium* | Inhibits H1N1 (IC_50_ >100 µg/mL), H9N2 (IC_50_ >100 µg/mL), | [3] |
| 3,4-Dimethoxy-2-hydroxyxanthone (**12**) | *Polygala karensium* | Inhibits H1N1 (IC_50_ >100 µg/mL), H9N2 (IC_50_ >100 µg/mL), | [3] |
| 1,7-Dihydroxy-4-methoxyxanthone (**13**) | *Polygala karensium* | Inhibits H1N1 (IC_50_: 28.4 µg/mL), H9N2 (IC_50_: 25.6 µg/mL), H1N1 (WT) (IC_50_: 9.3 µg/mL), H1N1 (H274Y) (IC_50_: 12.8 µg/mL) | [3] |
| 3,6-Dihydroxy-1,2-dimethoxyxanthone (**14**) | *Polygala karensium* | Inhibits H1N1 (IC_50_ >100 µg/mL), H9N2 (IC_50_ >100 µg/mL) | [3] |
| 1,3,7-Trihydroxyxanthone (**15**) | *Polygala karensium* | Inhibits H1N1 (IC_50_: 26.8 µg/mL), H9N2 (IC_50_: 24.8 µg/mL), H1N1 (WT) (IC_50_: 13.4 µg/mL), H1N1 (H274Y) (IC_50_: 9.1 µg/mL) | [3] |
| 1,2-Dimethoxy-4-hydroxyxanthone (**16**) | *Polygala karensium* | Inhibits H1N1 (IC_50_ >100 µg/mL), H9N2 (IC_50_ >100 µg/mL) | [3] |
| 1,2,3,5-Tetrahydroxyxanthone (**17**) | *Polygala karensium* | Inhibits H1N1 (IC_50_: 26.9 µg/mL), H9N2 (IC_50_: 19.8 µg/mL), H1N1 (WT) (IC_50_: 13.7 µg/mL), H1N1 (H274Y) (IC_50_: 10.8 µg/mL) | [3] |
| 7-Hydroxy-1-methoxyxanthone (**18**) | *Polygala karensium* | Inhibits H1N1 (IC_50_ >100 µg/mL), H9N2 (IC_50_ >100 µg/mL) | [3] |
| 1,5,8-Trihydroxy-3-methoxyxanthone (**19**) | *Swertia chirayita* | Inhibits HBsAg (IC_50_ > 4.53 mM), HBeAg (IC_50_ > 4.53 mM), HBV DNA replication (IC_50_ > 1.13 mM) | [7] |
|  | *Swertia mussotii* | Inhibits HepG2.2.15/HBsAg (IC_50_ > 0.98 mM), HBeAg (IC_50_: 350 µM), HBV DNA replication (IC_50_: 90 µM) | [8] |
|  | *Swertia Punicea* | Inhibition of HBsAg (IC_50_: 13 μg/mL) | [9] |
| 1-Hydroxy-3, 7-dimethoxyxanthone (**20**) | *Swertia chirayita* | Inhibits HBsAg (IC_50_ > 1.98 mM), HBeAg (IC_50_ > 1.98 mM), HBV DNA replication (IC_50_: 160 µM) | [7] |
| 6-Hydroxyethyl-1,3,7-trimethoxy-xanthone (**21**) | *Swertia elata* | Anti-TMV (21.4 % inhibitory at 20 μM) | [10] |
| 6-Acetyl-1,3,7-trimethoxyxanthone (**22**) | *Swertia elata* | Anti-TMV (28.8 % inhibitory at 20 μM) | [10] |
| Pulmonarxanthone B (**23**) | *Swertia elatas* | Anti-TMV (20.8 % inhibitory at 20 μM) | [10] |
| 1,5-Dihydroxy-3-(2-  oxopropyl)-6-methoxycarbonylxanthone (**24**) | *Swertia elata* | Anti-TMV (17.6 % inhibitory at 20 μM) | [10] |
| Bracthone A (**25**) | *Swertia elata* | Anti-TMV (18.4 % inhibitory at 20 μM) | [10] |
| Norbellidifolin (**26**) | *Swertia mussotii* | Inhibits HepG2.2.15/HBsAg (IC_50_: 0.77 mM), HBeAg (IC_50_ < 0.62 mM), HBV DNA replication (IC_50_: 20 µM) | [8] |
| 1,7-Dihydroxy-3,8-dimethoxyxanthone (**27**) | *Swertia mussotii* | Inhibits HBV DNA replication (IC_50_: 130 µM) | [8] |
| Swerpunilactone A (**28**) | *Swertia punicea* | Inhibits HepG2.2.15/HBsAg (IC_50_: 250 µM), HBeAg (IC_50_: 860 µM), HBV DNA replication (IC_50_: 180 µM) | [11] |
| Swerpunilactone B (**29**) | *Swertia punicea* | Inhibits HepG2.2.15/HBsAg (IC_50_: 290 µM), HBeAg (IC_50_: 310 µM), HBV DNA replication (IC_50_: 190 µM) | [11] |
| 2-Hydroxy-1-(hydroxymethyl)-8-methoxy-3-methyl-9*H*-xanthen-9-  one (**30**) | *Aspergillus sydowii* SCSIO 41301 from the sponge *Phakellia*  *fusca* | Inhibits A/PuertoRico/8/34 (H1N1) (IC_50_: 4.7 µM), A/Aichi/2/68 (H3N2) (IC_50_ >50 µM), A/FM-1/1/47 (H1N1) (IC_50_: 4.0 µM) | [12] |
| 2-Hydroxy-1-(hydroxymethyl)-7,8-dimethoxy-3-methyl-9*H*-xanthen-  9-one (**31**) | *Aspergillus sydowii* SCSIO 41301 from the sponge *Phakellia*  *fusca* | Inhibits A/PuertoRico/8/34 (H1N1) (IC_50_: 2.2 µM), A/Aichi/2/68 (H3N2) (IC_50_ >50 µM), A/FM-1/1/47 (H1N1) (IC_50_ >50 µM) | [12] |
| 3,8-Dihydroxy-2-methyl-9-oxoxanthene-4-carboxylic acid methyl ester (**32**) | *Fungus Penicillium* sp. SCSIO Ind16F01 | Inhibits H3N2 and EV71 viruses | [13] |
| 8-Hydroxy-6-methyl-9-oxo-9*H*-xanthene-1-carboxylic acid methyl ester (**33**) | *Fungus Penicillium* sp. SCSIO Ind16F01 | Inhibits H3N2 and EV71 viruses | [13] |
| 7,8-Dihydroxy-6-methyl-9-oxo-9*H*-xanthene-1-carboxylic acid methyl ester (**34**) | *Fungus Penicillium* sp. SCSIO Ind16F01 | Inhibits H3N2 and EV71 viruses | [13] |
| 2,3,6,8-Tetrahydroxy-1-methylxanthone (**35**) | *Fungus Wardomyces anomalus* | 82.2% Inhibitory of HIV-1-RT | [14] |
| 3,6,8-Trihydroxy-1-methylxanthone (**36**) | *Fungus Wardomyces anomalus* | 82.9% Inhibitory of HIV-1-RT | [14] |
|  | *Sponge-derived fungus Stachybotry sp.* HH1 ZDDS1F1-2 | Inhibits EV71 (IC_50_: 40.3 µM) | [15] |
| Methyl-(2-chloro-l,6-dihydroxy-3-methylxanthone)-8-carboxylate (**37**) | *Soil fungus Aspergillus iizukae* KL33 | Inhibits H1N1 (IC_50_: 133.4 µM), HSV-1 (IC_50_: 55.5 µM), HSV-2 (IC_50_: 175.5 µM) | [16] |
| Methyl-(4-chloro-l,6-dihydroxy-3-methylxanthone)-8-carboxylate (**38**) | *Soil fungus Aspergillus iizukae* KL33 | Inhibits H1N1 (IC_50_: 44.6 µM), HSV-1 (IC_50_: 21.4 µM), HSV-2 (IC_50_: 76.7 µM) | [16] |
| Methyl-(4-chloro-6-hydroxy-1-methoxy-3-methylxanthone)-8-carboxylate (**39**) | *Soil fungus Aspergillus iizukae* KL33 | Inhibits H1N1 (IC_50_ >200 µM), HSV-1 (IC_50_: 139.4 µM), HSV-2 (IC_50_ >200 µM) | [16] |
| Methyl-(6-hydroxy-1-methoxy-3-methylxanthone)-8-carboxylate (**40**) | *Soil fungus Aspergillus iizukae* KL33 | Inhibits H1N1 (IC_50_ >200 µM), HSV-1 (IC_50_: 157.7 µM), HSV-2 (IC_50_: 163.3 µM) | [16] |
| 4-Chloro-1,6-dihydroxy-3-methylxanthone-8-carboxylic acid (**41**) | *Soil fungus Aspergillus iizukae* KL33 | Inhibits H1N1 (IC_50_ >200 µM), HSV-1 (IC_50_: 183.3 µM), HSV-2 (IC_50_ >200 µM) | [16] |
| Calyxanthone (**42**) | *Soil fungus Aspergillus iizukae* KL33 | Inhibits H1N1 (IC_50_ >200 µM), HSV-1 (IC_50_: 144.4 µM), HSV-2 (IC_50_ >200 µM) | [16] |
| Methyl-(l,6-dihydroxy-3-methylxanthone)-8-carboxylate (**43**) | *Soil fungus Aspergillus iizukae* KL33 | Inhibits H1N1 (IC_50_: 140.4 µM), HSV-1 (IC_50_: 75.7 µM), HSV-2 (IC_50_: 95.4 µM) | [16] |
| **Prenylated Xanthones** |  |  |  |
| Blancoxanthone (**44**) | *Calophyllum blancoi* | Inhibits HCoV 229E (IC_50_: 3 µg/mL) | [17] |
| Pyranojacareubin (**45**) | *Calophyllum blancoi* | Inhibits HCoV 229E (IC_50_: 15 µg/mL) | [17] |
| Mangostin (**46**) | *Garcinia mangostana* | Inhibits HIV-1 (IC_50_: 5.1 µM) | [18] |
| γ-Mangostin (**47**) | *Garcinia mangostana* | Inhibits HIV-1 (IC_50_: 4.8 µM) | [18] |
| Allanxanthone C (**48**) | *Garcinia oligantha* | Anti-TMV (IC_50_: 200 µM) | [4] |
| 1,3-Dihydroxy-6-  methoxy-2,4-bis(3-methyl-2-buten-1-yl)-9*H*-xanthen-9-one (**49**) | *Garcinia oligantha* | Anti-TMV (IC_50_: 200 µM) | [4] |
| Nigrolineaxanthone K (**50**) | *Garcinia paucinervis* | Anti-TMV (IC_50_: 200 µM) | [6] |
| 5-*O*-Methylxanthone V1 (**51**) | *Garcinia paucinervis* | Anti-TMV (IC_50_: 82.4 µM) | [6] |
| Ananixanthone (**52**) | *Garcinia paucinervis* | Anti-TMV (IC_50_: 68.9 µM) | [6] |
| Cudraxanthone G (**53**) | *Garcinia paucinervis* | Anti-TMV (IC_50_: 52.8 µM) | [6] |
| Merguenone (**54**) | *Garcinia paucinervis* | Anti-TMV (IC_50_: 200 µM) | [6] |
| Biyouxanthone A (**55**) | *Hypericum chinense* | 89% Inhibitory of HCV at 10 µM | [19] |
| Biyouxanthone B (**56**) | *Hypericum chinense* | 61% Inhibitory of HCV at 10 µM | [19] |
| Macluraxanthone B (**57**) | *Maclura tinctoria* | Anti-HIV (EC_50_: 1-2 µg/mL) | [20] |
| Macluraxanthone C (**58**) | *Maclura tinctoria* | Anti-HIV (EC_50_: 1.3-2.2 µg/mL) | [20] |
| Gartanin (**59**) | *Maclura tinctoria* | Anti-HIV (EC_50_: >10 µg/mL) | [20] |
| 8-Desoxygartanin (**60**) | *Maclura tinctoria* | Anti-HIV (EC_50_: >10 µg/mL) | [20] |
| Pestalofone E (**61**) | *Pestalotiopsis fici* | Inhibits HIV-1 replication in C8166 cells (EC_50_: 93.7 µM) | [21] |
| Vieillardixanthone (**62**) | *Swertia elata* | Anti-TMV (15.2% inhibitory at 20 μM) | [10] |
| **Glycosylated Xanthones** |  |  |  |
| Mangiferin (**63**) | *Fridericia formosa* | Inhibits HSV-1 (EC_50_: 267.9 µg/mL), VACV-WR (EC_50_: 182.7 µg/mL), DENV-2 (EC_50_: 265.5 µg/mL) | [22] |
|  | *Mangifera indica* | Inhibits AR-29 (IC_50_: 2.9 µg/mL), KOS (IC_50_: 3.5 µg/mL) | [23] |
|  | *Swertia chirayita* | Inhibits HBsAg (IC_50_ > 3.03 mM), HBeAg (IC_50_ > 3.03 mM), HBV DNA replication (IC_50_ > 0.76 mM) | [9] |
| 2′-*O*-*Trans*-caffeoylmangiferin (**64**) | *Fridericia formosa* | Inhibits HSV-1 (EC_50_: 4.6 µg/mL), VACV-WR (EC_50_: 23.8 µg/mL), DENV-2 (EC_50_: 4.1 µg/mL) | [22] |
|  |  |  |  |
| 2′-*O*-*Trans*-coumaroylmangiferin (**65**) | *Fridericia formosa* | Inhibits HSV-1 (EC_50_: 47.4 µg/mL), EMCV (EC_50_: 241.0 µg/mL), DENV-2 (EC_50_: 40.4 µg/mL) | [22] |
| 2′-*O*-*Trans*-cinnamoylmangiferin (**66**) | *Fridericia formosa* | Inhibits HSV-1 (EC_50_: 77.4 µg/mL), DENV-2 (EC_50_: 3.5 µg/mL) | [22] |
| 1,7-Dihydroxyxanthone-6-*O*-*β*-D-glucoside (**67**) | *Mammea harmandii* | Anti-HIV1 effect | [24] |
| Mangiferin methyl ether (**68**) | *Mangifera indica* | 27.7 and 13.3 % Inhibitory of NA and CA-pro, respectively | [25] |
| Mangiferoxanthone A (**69**) | *Mangifera indica* | 55.8 and 46.1 % Inhibitory of NA and CA-pro, respectively | [25] |
| 2-*C*-*β*-D-Glucopyranosyl-1,3,7- trihydroxyxanthane (**70**) | *Swertia chirayita* | Inhibits HBsAg (IC_50_ > 2.15 mM), HBeAg (IC_50_ > 2.15 mM), HBV DNA replication (IC_50_ > 0.54 mM) | [7] |
|  | *Swertia mussotii* | Inhibits HepG2.2.15/HBsAg (IC_50_: 210 µM), HBeAg (IC_50_: 40 µM), HBV DNA replication (IC_50_: 90 µM) | [8] |
| 8-*O*-[*β*-D-Xylopyranosyl-(1→6)-*β*-D-glucopyranosyl]-1,7-dihydroxyl- 3-methoxyxanthone (**71**) | *Swertia chirayita* | Inhibits HBsAg (IC_50_ > 1.81 mM), HBeAg (IC_50_ > 1.81 mM), HBV DNA replication (IC_50_: 300 µM) | [7] |
| 8-*O*-[*β*-D-Xylopyranosyl-(1→6)-*β*-D-glucopyranosyl]-1-hydroxyl-3,7-dimethoxy-xanthone (**72**) | *Swertia chirayita* | Inhibits HBsAg (IC_50_: 2.56 mM), HBeAg (IC_50_ > 2.56 mM), HBV DNA replication (IC_50_: 70 µM) | [7] |
| 1-*O*-*β*-D-Glucopyranosyl-3,5,8-trihydroxyxanthone (**73**) | *Swertia chirayita* | Inhibits HBsAg (IC_50_: 400 µM), HBeAg (IC_50_ > 1.47 mM), HBV DNA replication (IC_50_ > 0.47 mM) | [7] |
|  | *Swertia mussotii* | Inhibits HBV DNA replication (IC_50_: 10 µM) | [8] |
| 7-*O*-[*β*-D-Xylopyranosyl-(1→2)-*β*-D-xylopyranosyl]-1,8-dihydroxy-3-methoxyxanthone (**74**) | *Swertia chirayita* | Inhibits HBsAg (IC_50_: 880 µM), HBeAg (IC_50_: 880 µM), HBV DNA replication (IC_50_: 220 µM) | [7] |
| 7-*O*-[*β*-D-xylopyranosyl-(1→2)-*β*-D-xylopyranosyl]-1,8-dihydroxy-3-methoxyxanthone (**74**) | *Swertia mussotii* | Inhibits HBV DNA replication (IC_50_: 100 µM) | [8] |
| Swertifrancheside (**75**) | *Swertia franchetiana* | Inhibits HIV-l (ED_50_: 30.9 *µ*g/mL) | [26] |
| Swertipunicoside (**76**) | *Swertia franchetiana* | Inhibits HIV-l (ED_50_: 3.0 µg/mL) | [26] |
| Norswertianin-1-*O*-*β*-D-glucoside (**77**) | *Swertia mussotii* | Inhibits HBV DNA replication (IC_50_: 60 µM) | [8] |
| Norswertianolin (**78**) | *Swertia mussotii* | Inhibits HBV DNA replication (IC_50_: 10 µM) | [8] |
| Swertianolin (**79**) | *Swertia Punicea* | Inhibition of HBeAg (IC_50_: 8.0 μg/mL) | [9] |
| **Caged Xanthones** |  |  |  |
| 7-Methoxydeoxymorellin (**80**) | *Garcinia hanburyi* | 23.2% Inhibitory of HIV -1 | [27] |
| 2-Isoprenylforbesione (**81**) | *Garcinia hanburyi* | 52.1% Inhibitory of HIV -1 | [27] |
| 8,8a-Epoxymorellic acid (**82**) | *Garcinia hanburyi* | Inhibits HIV-1 (IC_50_: 101.8 µg/mL) | [27] |
| Desoxymorellin (**83**) | *Garcinia hanburyi* | Inhibits HIV-1 (IC_50_: 186.2 µg/mL) | [27] |
| Isomorellin (**84**) | *Garcinia hanburyi* | 49.5% Inhibitory of HIV-1 | [27] |
| Isomorellinol (**85**) | *Garcinia hanburyi* | 42.2% Inhibitory of HIV-1 | [27] |
| Morellic acid (**86**) | *Garcinia hanburyi* | Inhibits HIV-1 (IC_50_: 11.0 µg/mL) | [27] |
| Gambogic acid (**87**) | *Garcinia hanburyi* | Inhibits HIV-1 (IC_50_: 15.0 µg/mL) | [27] |
| Desoxygambogenin (**88**) | *Garcinia hanburyi* | 64.0% Inhibitory of HIV-1 | [27] |
| Hanburin (**89**) | *Garcinia hanburyi* | Inhibits HIV-1 (IC_50_: 190.7 µg/mL) | [27] |
| Forbesione (**90**) | *Garcinia hanburyi* | Inhibits HIV-1 (IC_50_: 62.1 µg/mL) | [27] |
| Dihydroisomorellin (**91**) | *Garcinia hanburyi* | Inhibits HIV-1 (IC_50_: 42.3 µg/mL) | [27] |

**References**

1. Luo X, Yang J, Chen F, Lin X, Chen C, Zhou X, Liu S, Liu Y. Structurally diverse polyketides from the mangrove-derived fungus Diaporthe sp. SCSIO 41011 with their anti-influenza A virus activities. Frontiers in Chemistry. 2018 Jul 12;6:282.
2. Zhang H, Tao L, Fu WW, Liang S, Yang YF, Yuan QH, Yang DJ, Lu AP, Xu HX. Prenylated benzoylphloroglucinols and xanthones from the leaves of Garcinia oblongifolia with antienteroviral activity. Journal of natural products. 2014 Apr 25;77(4):1037-46.
3. Dao TT, Dang TT, Nguyen PH, Kim E, Thuong PT, Oh WK. Xanthones from Polygala karensium inhibit neuraminidases from influenza A viruses. Bioorganic & medicinal chemistry letters. 2012 Jun 1;22(11):3688-92.
4. Wu YP, Zhao W, Xia ZY, Kong GH, Lu XP, Hu QF, Gao XM. Three new xanthones from the stems of Garcinia oligantha and their anti-TMV activity. Phytochemistry Letters. 2013 Nov 1;6(4):629-32.
5. Meng YanLin MY, Yang YuChun YY, Qin Ying QY, Xia CongFang XC, Ye YanQing YY, Hu QiuFen HQ, Li YinKe LY. A new xanthone from the stems of Garcinia oligantha and their anti-tobacco mosaic virus activity.
6. Wu YP, Zhao W, Xia ZY, Kong GH, Lu XP, Hu QF, Gao XM. Three novel xanthones from Garcinia paucinervis and their anti-TMV activity. Molecules. 2013 Aug 13;18(8):9663-9.
7. Wu YP, Zhao W, Xia ZY, Kong GH, Lu XP, Hu QF, Gao XM. Three novel xanthones from Garcinia paucinervis and their anti-TMV activity. Molecules. 2013 Aug 13;18(8):9663-9.
8. Cao TW, Geng CA, Ma YB, He K, Wang HL, Zhou NJ, Zhang XM, Tao YD, Chen JJ. Xanthones with anti-hepatitis B virus activity from Swertia mussotii. Planta Medica. 2013 May;79(08):697-700.
9. Zhang XQ, Chen JC, Huang FJ, Tian LY, Tu Y. Anti-HBV activities of xanthones from Swertia Punicea Hemsl. North American Journal of Medicine and Science. 2014 Jul 31;7(2).
10. Jiang W, Zhu DL, Wang MF, Yang QS, Zuo MY, Zeng L, Li GP. Xanthones from the herb of Swertia elata and their anti-TMV activity. Natural Product Research. 2016 Aug 17;30(16):1810-5.
11. Wang HL, Cao TW, Jiang FQ, Geng CA, Zhang XM, Huang XY, Wang LJ, Liang WJ, Rong GQ, Chen JJ. Swerpunilactones A and B, the first example of xanthone and secoiridoid heterodimers from Swertia punicea, S. hispidicalyx, and S. yunnanensis. Tetrahedron Letters. 2013 May 22;54(21):2710-2.
12. Liu N, Peng S, Yang J, Cong Z, Lin X, Liao S, Yang B, Zhou X, Zhou X, Liu Y, Wang J. Structurally diverse sesquiterpenoids and polyketides from a sponge-associated fungus Aspergillus sydowii SCSIO41301. Fitoterapia. 2019 Jun 1;135:27-32.
13. Liu FA, Lin X, Zhou X, Chen M, Huang X, Yang B, Tao H. Xanthones and quinolones derivatives produced by the deep-sea-derived fungus Penicillium sp. SCSIO Ind16F01. Molecules. 2017 Dec 7;22(12):1999.
14. Abdel-Lateff A, Klemke C, König GM, Wright AD. Two new xanthone derivatives from the algicolous marine fungus Wardomyces anomalus. Journal of Natural Products. 2003 May 23;66(5):706-8.
15. Qin C, Lin X, Lu X, Wan J, Zhou X, Liao S, Tu Z, Xu S, Liu Y. Sesquiterpenoids and xanthones derivatives produced by sponge-derived fungus Stachybotry sp. HH1 ZSDS1F1-2. The Journal of Antibiotics. 2015 Feb;68(2):121-5.
16. Kang HH, Zhang HB, Zhong MJ, Ma LY, Liu DS, Liu WZ, Ren H. Potential anti-viral xanthones from a coastal saline soil fungus Aspergillus iizukae. Marine drugs. 2018 Nov 15;16(11):449.
17. Shen YC, Wang LT, Khalil AT, Chiang LC, Cheng PW. Bioactive pyranoxanthones from the roots of Calophyllum blancoi. Chemical and pharmaceutical bulletin. 2005;53(2):244-7.
18. Chen SX, Wan M, Loh BN. Active constituents against HIV-1 protease from Garcinia mangostana. Planta medica. 1996 Aug;62(04):381-2.
19. Tanaka N, Mamemura T, Abe S, Imabayashi K, Kashiwada Y, Takaishi Y, Suzuki T, Takebe Y, Kubota T, Kobayashi JI. Biyouxanthones A-D, prenylated xanthones from roots of Hypericum chinense. Heterocycles. 2010 Jan 1;80(1):613.
20. Groweiss A, Cardellina JH, Boyd MR. HIV-Inhibitory Prenylated Xanthones and Flavones from Maclura t inctoria. Journal of natural products. 2000 Nov 27;63(11):1537-9.
21. Liu L, Liu S, Chen X, Guo L, Che Y. Pestalofones A–E, bioactive cyclohexanone derivatives from the plant endophytic fungus Pestalotiopsis fici. Bioorganic & Medicinal Chemistry. 2009 Jan 15;17(2):606-13.
22. Brandão GC, Kroon EG, Souza Filho JD, Oliveira AB. Anti-viral activity of Fridericia formosa (Bureau) LG Lohmann (Bignoniaceae) extracts and constituents. Journal of tropical medicine. 2017;2017(1):6106959.
23. Rechenchoski DZ, Agostinho KF, Faccin-Galhardi LC, Lonni AA, da Silva JV, de Andrade FG, Cunha AP, Ricardo NM, Nozawa C, Linhares RE. Mangiferin: a promising natural xanthone from Mangifera indica for the control of acyclovir–resistant herpes simplex virus 1 infection. Bioorganic & Medicinal Chemistry. 2020 Feb 15;28(4):115304.
24. Liangsakul P, Kuhakarn C, Hongthong S, Jariyawat S, Suksen K, Akkarawongsapat R, Limthongkul J, Napaswad C, Reutrakul V. Anti-HIV 1 Activity of Xanthones from the Bark of Mammea harmandii. Natural Product Communications. 2018 Jan;13(1):1934578X1801300116.
25. Abdel-Mageed WM, Bayoumi SA, Chen C, Vavricka CJ, Li L, Malik A, Dai H, Song F, Wang L, Zhang J, Gao GF. Benzophenone C-glucosides and gallotannins from mango tree stem bark with broad-spectrum anti-viral activity. Bioorganic & medicinal chemistry. 2014 Apr 1;22(7):2236-43.
26. Wang JN, Hou CY, Liu YL, Lin LZ, Gil RR, Cordell GA. Swertifrancheside, an HIV-reverse transcriptase inhibitor and the first flavone-xanthone dimer, from Swertia franchetiana. Journal of natural products. 1994 Feb;57(2):211-7.
27. Reutrakul V, Anantachoke N, Pohmakotr M, Jaipetch T, Sophasan S, Yoosook C, Kasisit J, Napaswat C, Santisuk T, Tuchinda P. Cytotoxic and anti-HIV-1 caged xanthones from the resin and fruits of Garcinia hanburyi. Planta medica. 2007 Jan;73(01):33-40.
